# Motives relate to cooperation in social dilemmas but have an inconsistent association with leadership evaluation

**DOI:** 10.1038/s41598-019-45931-4

**Published:** 2019-07-12

**Authors:** Christian Wolff, Nina Keith

**Affiliations:** 1 Work and Organizational Psychology, University of Bamberg, Bamberg, Germany; 2 Organizational and Business Psychology, Technische Universität Darmstadt, Darmstadt, Germany

**Keywords:** Human behaviour, Psychology and behaviour

## Abstract

A common assumption is that good leaders are driven by a power motive that motivates them to influence others. However, leaders need to restrain themselves in social dilemmas where cooperation maximizes collective outcomes. We theorize that in social dilemmas, a desire for positive relationships (affiliation motive) is more beneficial than a power motive because it draws attention away from short-term self-interest towards understanding others. In a game of *Settlers of Catan* in the laboratory, we find that a functional variant of the affiliation motive relates to verbal encouragement of cooperation, to fewer occurrences of oil spills, to higher ratings of transformational leadership and, in a field survey, to fewer selfish business decisions. Furthermore, a dysfunctional variant of the power motive relates to two of three indicators of selfishness. Group members perceive selfish individuals as assuming leadership roles which indirectly relates to slightly higher ratings of transformational leadership. This pattern of evaluation may privilege men who, on average, show more selfish behaviour which can be partially attributed to their motives. Mere awareness of gender-based discrimination does not enable raters to circumvent this pattern of evaluation. This work suggests a need for interventions that increase appreciation of cooperative leaders.

## Introduction

Mastering social dilemmas is essential. Humans achieve many kinds of progress through cooperation in social dilemmas^1,2^, such as when business departments share their knowledge to create a useful product^3^. In social dilemmas, each party is best off in the short term by minimizing their personal costs^4^ whereas in the long term, wisely chosen cooperation can pay off way beyond its initial costs^5^ and increases prosperity.

In social dilemmas, leader behaviour matters. Leaders often have discretion to choose a course of action^6,7^. Different leaders make different choices^8^. Here we argue that leaders’ choices are affected by their motives. Motives are stable preferences for particular classes of states or activities. Leaders’ behaviour in social dilemmas is of high importance for their organizations even beyond the scope of a single situation^9^. It sparks lasting reciprocity from stakeholders and observers^10^, signals trustworthiness^11^, builds reputation^5^, and maintains existing relationships. Moreover, leaders serve as role models for followers who imitate them and who are inspired by leaders with integrity^12^.

Motives shape how people understand social dilemmas. Resolving social dilemmas requires an unbiased understanding of the situation^13,14^. Actors need to (*i*) recognize interdependencies in the distribution of everyone’s outcomes and (*ii*) anticipate what others will do^15^. Empathy (being able to perceive others’ mental states^16^) allows both—an appraisal of dilemma outcomes from the perspective of others^17–20^ and, based on that, the drawing of inferences about others’ intentions^17^. Empathy itself depends heavily on motivation^17^. Some motives have excitatory or inhibitory effects on it. In this way, motives can determine behaviour in social dilemmas.

### The affiliation motive and cooperation

Here we first propose that an affiliation motive relates positively to cooperation. The affiliation motive refers to a desire to build and maintain positive relationships. Affiliation attracts people to situations in which they can connect with others^17^ and motivates them to attend to others’ mental states^17^. In social dilemmas, attending to others enables an accurate understanding of the situation, which in turn fosters cooperation^18–22^.

However, we limit this proposition to a *functional* variant of the affiliation motive. We theorize that those high in this variant particularly enjoy being considerate and cooperative rather than being popular or being validated by others. This conceptualization deviates from prior research, which often cast affiliation in a negative light^23,24^. Affiliation motivated individuals have often been seen as desperately wanting to be liked, fearing rejection, avoiding conflicts, and favoring their in-group at the expense of everyone else^23^. In this study, we refer to this as the *dysfunctional* affiliation motive and include it only for comparison. We do not expect the dysfunctional affiliation motive to relate to caring and trusting nor, in turn, to cooperation. We use the terms *functional* and *dysfunctional* to allude to the assumed implications that each motive variant was theorized to have for social interactions, especially in the context of leadership^23,25–28^.

### The power motive and selfishness

Second, we propose that a power motive relates positively to selfishness. The power motive refers to the desire to influence or control people or processes. Experiencing power heightens sensitivity for rewards^29^, narrows focus of attention by suppressing constraining information^30^, makes people play down risks^31^, and increases overconfident decisions^32^. In social dilemmas, this should direct individuals toward selfish choices, which typically provide the most salient rewards. Experiencing power particularly affects the processing of social information. That is, power can decrease taking others’ perspectives^33^ or their advice^34^ and sometimes undermines coordination with others^35^. People who are motivated to pursue self-interest often reduce empathy^17^. In this way, a strong power motive may deter individuals from recognizing how cooperation benefits everyone in the long run and what is wrong with a selfish choice. This may cause biased understandings of social dilemmas, which in turn lead to selfish behaviour.

However, we limit this proposition to a *dysfunctional* variant of the power motive. Prior research has shown that humans desire power for various purposes^23,36,37^. We theorize that those high in the dysfunctional variant of the power motive desire power as a means to perceived superiority in an authoritarian or materialistic sense. In contrast, if an individual desires power in order to pursue a greater good, we refer to that as a *functional* power motive and include it in this study only for comparison. We do not expect the functional power motive to relate to selfishness because its other-related purpose should compensate negative effects of being motivated by power^38^.

### Previous research on motives and cooperation in social dilemmas

We believe that the present work contributes to the literature on cooperation in social dilemmas by using an alternative approach to measuring motives. For half a century now, a rich body of literature has accumulated^14,39,40^ showing that cooperation in social dilemmas can be predicted from *social value orientation* (SVO) both in the laboratory^41^ as well as in several field studies^42,43^. Researchers measure SVO by asking participants to split money between themselves and a fictional stranger (called decomposed games approach). Researchers then derive a score classifying participants as prosocial, individualistic, competitive, or unclassifiable^44,45^. This approach infers participants’ social preferences indirectly from their choices.

Here we use a different approach based on self-reported motives^46^. We consider two separate motive variants at once (functional affiliation motive, dysfunctional power motive). While the functional affiliation motive emphasizes on a concern for others’ interests, the dysfunctional power motive focuses on self-enhancement through devaluation of others. Examining both motives simultaneously may help gauging each motive’s relative importance for cooperation in social dilemmas. Furthermore, being able to distinguish these motive variants from related motive variants (dysfunctional affiliation motive, functional power motive) may sharpen our understanding of the boundaries of each motive variant. Previous research has already predicted cooperation from personality^14,47^ (which can be seen as closely related to motives^48^) and from values or motives^21,46,49–51^. However, the relative importance of multiple motives for cooperation in social dilemmas seems less clear (but see refs^21,46^).

### Previous research on motives and leadership

Furthermore, we hope that the present study contributes to the literature on the role of motives for leadership. Previous research has focused primarily on *implicit* (i.e., subconsciously activated) motives. Supplementary Table S1 provides an overview of all studies on the role of implicit affiliation and power motives for leadership or leader outcomes that we are aware of (*k* = 26 samples, *n* = 2,495 participants). This overview suggests that it is difficult to draw any overall conclusions from these studies as a whole. Whereas an early study indicated that a *low* implicit affiliation motive might be beneficial in leaders^52^, almost all of the other studies yielded contradictory^53–56^ or inconsistent^36,57–61^ results—including a reanalysis of data from the original sample^62^. Supplementary Table S1 also suggests that it is difficult to draw conclusions about the role of the implicit power motive for effective leadership. As presented in Columns 11 and 12 of Supplementary Table S1, most studies examined a specific variant of the implicit power motive (15 samples^36,52,55,56,59,62–66^) or its combination with other motives (4 samples^54,57,58^). While doing so, researchers used a total of 10 different operationalizations^36,52,55,57,59,63,64,66^ (cf. Columns 11 and 12 in Supplementary Table S1). This degree of heterogeneity makes it difficult to interpret individual studies or to integrate findings across multiple studies. It may also explain why motives were only included in 2 of the 15 reviews and meta-analyses on the role of individual differences for leadership since 2011^67^. Both reviews did not systematically synthesize empirical research^28,68^ and one did not distinguish between functional and dysfunctional variants of motives^68^.

Here we focus on *explicit* (i.e., consciously accessible) affiliation and power motives and include *implicit* motives only as control variables in one sample. We can thereby examine if theoretical assumptions about the role of affiliation and power motives for leadership^23,26,28^ apply to the *explicit* motivational system. Dual motive theory postulates that implicit and explicit motives belong to two separate motivational systems^69–71^. While implicit motives are assumed to energize *operant* behaviour which is spontaneously enacted and driven by affect, explicit motives are expected to influence *respondent* behaviour which is subject of conscious thought and deliberation^72^. Leadership roles may often require individuals to think through decisions and to consider factors that are imposed from outside. Both of these processes are theorized to be influenced by the explicit motivational system^72^. A large body of literature supports the importance of explicit variables^73^ and explicit motives^74^ for job performance^75^ and effective leadership^62,76^. However, we know of no measures that enable the assessment of variants of explicit affiliation and power motives as they are conceptualized in the literature^23,26–28,77^ (for existing scales, see refs. ^37,78,79^). For this reason, we developed short self-report scales that measure functional and dysfunctional variants of affiliation and power motives. These scales provide an opportunity to study the importance of explicit motive variants for leadership outcomes.

### Gender differences in motives and cooperation

Finally, we expect that men show more selfish behaviour than women and that this can be partially attributed to gender differences in motives. We know from meta-analyses that men, on average, have lower moral sensitivity than women^80^, exhibit weaker deontological inclinations^81^, and behave more selfishly in resource dilemmas^82^. Even though there are detailed theoretical accounts for gender differences and similarities in general^83,84^, scholars still call for more research on the specific factors that give rise to gender differences^85^.

We draw on sociocultural theory^84^ as an explanation for gender differences in motives. Based on historical division of labour by gender, women’s assignment to the role of child care might have contributed to the development of a higher functional affiliation motive in women because of the congruency between this motive and the responsibilities associated with child care^86^. Previous findings from large-sampled studies appear to support this idea. Women place, on average, higher importance than men on values that are directed towards the well-being of others such as benevolence, universalism^87^, and social values in general^88,89^. Other research shows that sociocultural factors also underlie gender differences in competition^90^ which may be intended to preserve the gender hierarchy^91,92^. This might have contributed to the development of a higher dysfunctional power motive in men who, on average, place more value on control than women^93^, respond more strongly to intergroup conflicts^94^, and experience competition more positively^95^. Based on our propositions about the roles of the functional affiliation motive and the dysfunctional power motive for cooperation, we assume that gender differences in these motives translate to gender differences in cooperation.

### The present study

To test our propositions, we observed groups during a game of *Settlers of Catan*. In this game, players need to make efficient use of resources in order to populate an uninhabited island. All players manage their own population. Players can compete or cooperate at any given time. We choose the *Oil Springs* iteration of this game, which creates a resource dilemma by providing the option to use oil. Using oil allows players to extend their empire, but gradually destroys the island and its resources^96^. We set financial incentives that were intended to activate motives related to both cooperation and selfishness. Participants knew that after the game, a coin toss determined whether they received a payment based on group performance (intended to activate motives related to cooperation) or on individual performance (intended to activate motives related to selfishness). A total of 201 individuals participated in groups of 3 to 4 players who hardly knew each other before meeting in the laboratory.

During the game, which lasted about 75 min, we videotaped the whole conversation. Communication about a dilemma often increases cooperation rates^13^. As a measure of verbal encouragement of cooperation, we count and aggregate all statements that favor either cooperation (e.g., “let us avoid using oil”) or selfishness (e.g., “everyone should look out for themselves”, inverse coded).

## Results

### Motives relate to cooperation in social dilemmas

**Figure 1:**
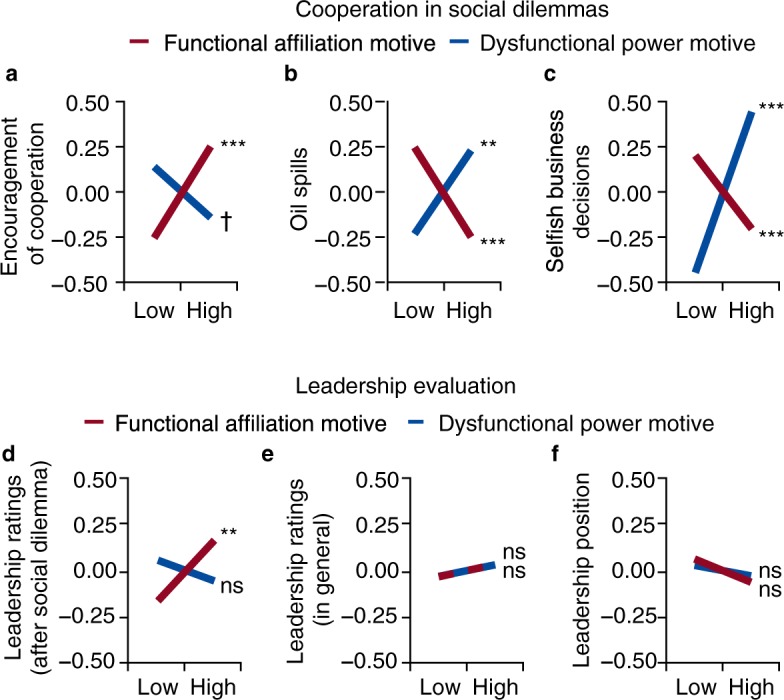
Motives relate to cooperation in social dilemmas but have little impact on leadership evaluations. (**a**,**b**) Motives relate to behaviour during a game of *Settlers of Catan* (*n* = 201). Conversations are videotaped and any statements favoring cooperation (positive values) or selfishness (negative values) are counted, log-transformed, aggregated using equal weights, and then aggregated over two independent observers (*r* = 0.71). By using oil, players cause oil spills, which damage the fictitious island of Catan. We count how many oil spills each player causes. (**c**) Motives relate to selfish decisions in a field survey (*n* = 960). Respondents read six business scenarios each posing a social dilemma. (**d**) After the game of Settlers of Catan, players rate each other on transformational leadership. (**e**) In the field survey, 739 peers rate the general leadership competence of 486 respondents. (**f**) Respondents state whether they hold a professional leadership position. All values on *y* axes are *z*-standardized. Lines represent slopes from multiple regression analysis while controlling for a dysfunctional affiliation motive and a functional power motive (Supplementary Tables S3, S4). Low/high ± 1 s.d. ^***^P < 0.001, ^**^P < 0.01, ^†^*P* < 0.10, two-sided *t*-tests. ns, not significant.

Figure 1a shows that the functional affiliation motive is positively related to encouragement of cooperation, *β* = 0.25, *P* = 0.0009, whereas the dysfunctional power motive is not significantly related to encouragement of cooperation, *β* = −0.14, *P* = 0.054.

We also count how many oil spills an individual causes during the game. Oil spills typically inflict lasting damage to one or more group members. We find that the functional affiliation motive is related to fewer occurrences of oil spills, *β* = −0.25, *P* = 0.0009, whereas the dysfunctional power motive is related to more oil spills, *β* = 0.23, *P* = 0.0013 (Fig. 1b).

As another test of both propositions, we conducted a field survey. We recruited 961 individuals online (*M*_age_ = 31 y, s.d. = 12) who read six business scenarios. Each scenario describes a social dilemma requiring a decision between personal benefits and preventing harm to society or the environment. Figure 1c shows that the functional affiliation motive is negatively related to selfish business decisions, *β* = −0.20, *P* < 0.0001, whereas the dysfunctional power motive is positively related to selfish business decisions, *β* = 0.44, *P* < 0.0001. Many respondents of this study are in leadership positions (*n* = 257) or have other kinds of work experience (*n* = 446). For all subsamples, similar results emerge (see Supplementary Table S2). This suggests that with regard to selfish business decisions, our results seem to generalize across different occupational statuses.

In both studies, all relationships involving the functional affiliation motive remain significant after we account for important control variables (e.g., personality traits, other motives, reasoning ability, Supplementary Fig. S1, Supplementary Table S3). This indicates that the functional affiliation motive accounts for aspects of human behaviour in the investigated social dilemmas beyond the predictive validity of established traits. Furthermore, we include a dysfunctional variant of the affiliation motive and a functional variant of the power motive in all analyses but find no substantial relationships to the outcomes so far described (Supplementary Table S3). Full correlation matrices are provided in Supplementary Datasets 1 and 2.

### Gender differences in motives and cooperation

**Figure 2:**
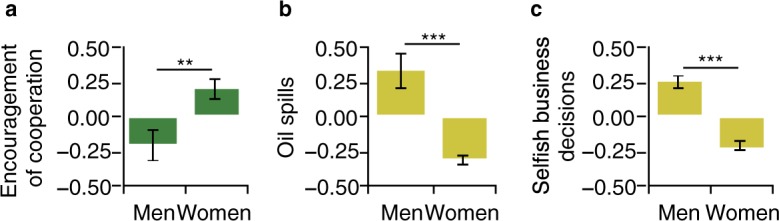
On average, women cooperate more than men. (**a**) Women (*n* = 103) encourage cooperation more consistently than men (*n* = 98), *d* = 0.40. (**b**) Men are 583% more likely to cause an oil spill than women (39 vs. 6), *d* = −0.67. (**c**) Men’s (*n* = 448) decisions in business scenarios are more selfish than women’s (*n* = 512), *d* = −0.47. Bars represent *z*-standardized means ± 1 s.e.m. Raw values are presented in Supplementary Table S5. ^***^P < 0.001, ^**^P < 0.01, two-sided *t*-tests.

**Figure 3:**
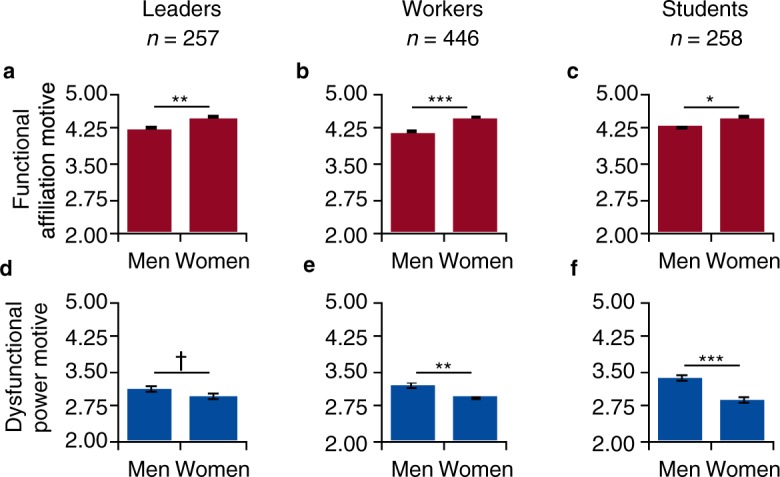
Across occupational groups, women report a stronger functional affiliation motive and men report a stronger dysfunctional power motive. (**a**,**d**) Respondents state whether they currently hold a professional leadership position or, if they are not working anymore, held one in the past. (**b**,**e**) Respondents without leadership position but with work experience (3 mo to 52 y, mean = 9 y, s.d. = 11). (**c**,**f**) Respondents are mostly students or homemakers (mean age = 22 y, s.d. = 5). All data are from the field survey (*n* = 961). Scales range from 1 “does not apply at all” to 6 “fully applies.” Bars represent means ± 1 s.e.m. ^***^P < 0.001, ^**^P < 0.01, ^*^P < 0.05, ^†^*P* < 0.10, two-sided *t*-tests.

Next, we examine gender differences in cooperation and in the motives underlying it. If such analyses highlight strengths that are—on average—associated with the female gender, a finding like this might contribute towards deconstructing male leadership stereotypes^97,98^. Figure 2 shows that women encourage cooperation more consistently than men, *d* = 0.40, *P* = 0.006, whereas men are more than 500% more likely to cause an oil spill (39 of 45 are caused by men), *d* = −0.67, *P* < 0.0001, and make business decisions that are more selfish, *d* = −0.47, *P* < 0.0001 (see Supplementary Table S5 for more detail). These gender differences in cooperative behaviour can partially be attributed to gender differences in motives. More specifically, women report a stronger functional affiliation motive than men in both the laboratory (*d* = 0.64) and field study (*d* = 0.39) whereas men report a stronger dysfunctional power motive than women (*d*s = −0.49 and −0.32, respectively). Gender differences in these motives are largely consistent across different occupational statuses (Fig. 3, Supplementary Table S5) with five of six effect sizes reaching statistical significance. Indirect effects of gender on cooperation via motives are reported in Supplementary Table S6.

### Motives hardly affect leadership evaluation

How do motives relate to leadership evaluations from others? On the one hand, those motivated to cooperate should receive *positive* evaluations given that they cooperate more and cooperation tends to be effective in our studies (e.g., cooperation enables high group performance in the game of Settlers of Catan, Supplementary Information, Section 4; Supplementary Fig. S3). On the other hand, those motivated to cooperate might receive *negative* evaluations because people hold stereotypes about leadership that expect leaders to be dominant rather than cooperative^97,99,100^ (Supplementary Information, Section 7). One way to express dominance in social dilemmas is through selfish behaviour such as causing oil spills.

We assess three types of leadership evaluation (Fig. 1d–f). In the laboratory, each player rates all other group members in transformational leadership after the game of Settlers of Catan, resulting in a total of 582 ratings (each ratee receives 2 to 3 ratings from group members—depending on group size—which we then average within each ratee). In an attempt to make sure that ratings are based specifically on behaviour during the game, we control for baseline ratings from before the game. We find that individuals with a strong functional affiliation motive tend to receive slightly higher ratings from their group members, *β* = 0.17, *P* = 0.0024 (Fig. 1d). In contrast, the dysfunctional power motive is unrelated to ratings of transformational leadership, *β* = −0.06, *P* = 0.28, despite its positive relationship to the occurrence of oil spills. In the field survey, 739 peers rate the general leadership competence of 486 respondents (*M* = 1.52 peer-ratings per respondent, s.d. = 0.75). Here we obtain null results for both motives (Fig. 1e). Neither the functional affiliation motive, *β* = 0.03, *P* = 0.47, nor the dysfunctional power motive, *β* = 0.03, *P* = 0.50, matter (despite their relationship with selfish business decisions). Instead, a functional power motive is important, *β* = 0.25, *P* < 0.0001 (Supplementary Table S4). We obtain similar results with respect to the occupancy of a professional leadership position (Fig. 1f). Again, neither the functional affiliation motive, *β* = −0.06, *P* = 0.074, nor the dysfunctional power motive, *β* = −0.03, *P* = 0.40, matter. Instead, a functional power motive is important, *β* = 0.21, *P* < 0.0001. In line with raters’ partial indifference towards selfishness, women do not, on average, receive more positive ratings than men after the game of Settlers of Catan, *d* = −0.06, *P* = 0.67, despite behaving more cooperatively on average (Supplementary Table S5).

**Figure 4:**
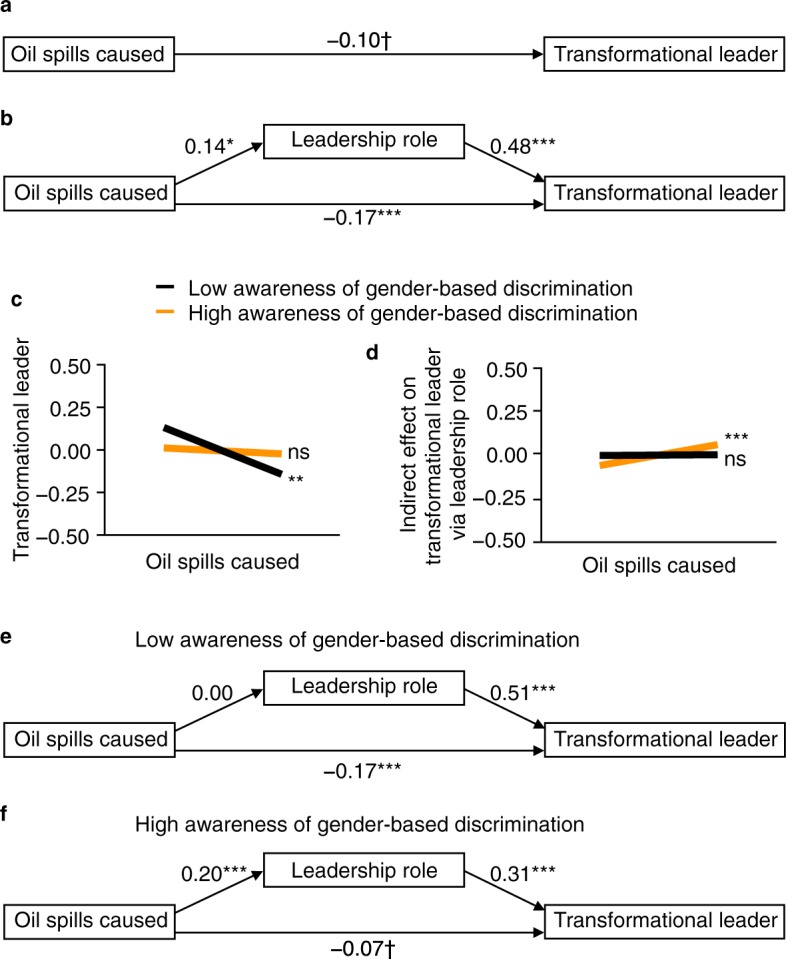
Perceivers think that selfish players assume a leadership role, which relates positively to their ratings of transformational leadership. (**a**) Overall, no substantial relationship exists between the number of oil spills an individual causes and the ratings of transformational leadership that individual receives from his/her group members after the game of *Settlers of Catan*, even though oil spills are very harmful. (**b**) Perceivers think that selfish players assume a leadership role, which in turn relates positively to leadership ratings and offsets more negative evaluations of oil spills (*n* = 201 players). (**c**–**f**) Awareness of gender-based discrimination itself promotes this stereotypical pattern of evaluation (*n* = 582 dyads, displayed are conditional effects). (**c**,**e**) Only group members with *low* (−1 s.d.) awareness of gender-based discrimination disapprove of (predominantly male) players who cause oil spills. (**d**,**f**) In contrast, group members with *high* (+1 s.d.) awareness of gender-based discrimination rate (predominantly male) players who cause oil spills as assuming a leadership role and, in turn, as transformational leaders. All coefficients are *z*-standardized. ^***^P < 0.001, ^**^P < 0.01, ^*^P < 0.05, ^†^*P* < 0.10, two-sided *t*-tests. ns, not significant.

Why do raters not always appreciate cooperativeness? After the game of Settlers of Catan, players also rate if they think that other players assumed a leadership role. We find that selfish players (those who cause oil spills) tend to be perceived as assuming a leadership role, *β* = 0.14, *P* = 0.015. Being perceived as assuming a leadership role, in turn, relates positively to ratings of transformational leadership, *β* = 0.48, *P* < 0.0001, so that a small indirect effect of oil spills to transformational leadership (via assumed leadership role) emerges, *β* = 0.069, *z* = 2.35, *P* = 0.019, 95% CI [0.007, 0.147] (Fig. 4b). This indirect effect somewhat offsets negative evaluations of oil spills, *β* = −0.17, *P* = 0.0002, so that, overall, no strong or significant relationship exists between oil spills and ratings of transformational leadership, *β* = −0.10, *P* = 0.057 (Fig. 4a), despite the havoc that oil spills are causing.

Exploratory analyses indicate that only some raters show this pattern of evaluation. More specifically, it is those with *high* awareness of gender-based discrimination (conditional effects for +1 s.d.) who do not seem to particularly appreciate cooperators. Instead, they tend to rate oil-spill causing group members—of which 84% are male—as assuming a leadership role, *β* = 0.20, *P* < 0.0001 (Fig. 4f), which results in a small positive indirect effect on transformational leadership, *β* = 0.061, s.e.m. = 0.019, 95% CI [0.028, 0.103] (Fig. 4d). In contrast, those with *low* awareness of gender-based discrimination (conditional effects for −1 s.d.) do not rate oil-spill causing group members as assuming a leadership role, *β* = 0.00, *P* = 0.92 (Fig. 4e), so that we find no significant indirect effect on transformational leadership, *β* = 0.002, s.e.m. = 0.035, 95% CI [−0.071, 0.065] (Fig. 4d). Instead, these raters with *low* awareness of gender-based discrimination tend to rate oil-spill causing group members as low on transformational leadership, *β = *−0.17, *P* = 0.0001 (total effect = −0.14, *P* = 0.004) which raters with *high* awareness of gender-based discrimination do not seem to do to a statistically significant extent, *β* = −0.07, *P* = 0.063 (total effect = −0.02, *P* = 0.68; Fig. 4c; Supplementary Information, Section 5; see Supplementary Information, Section 7 for a discussion of these results).

## Discussion

The present work examines the role of motives for cooperation and finds that a functional affiliation motive positively relates to cooperation and that a dysfunctional power motive negatively relates to two of three indicators of cooperation. On average, women cooperate more than men and this can be partially attributed to women’s higher functional affiliation motive and their lower dysfunctional power motive. All results involving the functional affiliation motive are consistent across dependent variables, across subsamples with different occupational statuses (including leaders), and after accounting for a number of relevant control variables (including motivation to lead, fairness, reasoning, and implicit motives). Results involving the dysfunctional power motive are less consistent after accounting for relevant control variables. When interpreting these findings, readers should keep in mind limitations such as the short length of the motive measures and their suboptimal psychometric properties (discussed below and in Supplementary Information, Section 2).

### Motives and cooperation in social dilemmas

We believe that this article adds information to the literature on social dilemmas. The present study uses a different approach to measuring motives than most studies on social dilemmas^41,45^. This may promote further insights into the nature of the motive variants that are relevant to cooperation in social dilemmas. We find that a concern for others’ interests (functional affiliation motive) relates to cooperation independently of a focus on self-enhancement through devaluation of others (dysfunctional power motive). Findings involving the dysfunctional power motive are less robust than those for the functional affiliation motive. These motive variants must not be confused with a striving for harmonious relationships (dysfunctional affiliation motive) or a desire to pursue power for the greater good (functional power motive) which we control in our study, thereby delineating a boundary condition of our propositions^101^. We believe that this approach clarifies a detail about the specific nature of the motives that relate to cooperation in social dilemmas.

### Motives and effective leadership

We assume that the present work contributes to the literature on the role of motives for leadership. A functional affiliation motive seems to be desirable in leaders whereas a dysfunctional power motive tends to be undesirable—at least if one considers cooperation to be essential for effective leadership^8,12,102–104^. So far, affiliation and power motives were left out of most of the recent reviews and meta-analyses on the role of individual differences for leadership^67^. To the best of our knowledge, there has been no definitive answer which motive variants are desirable in leaders^23,36,52–66^ (cf. Supplementary Table S1 for an overview of previous studies on the role of implicit affiliation and power motives for leadership). Maybe more research is needed that clarifies the roles of affiliation and power motives (both implicit and explicit) for different criteria of effective leadership such as ratings or cooperation. The present study may contribute to this endeavour. It introduces an economic alternative to previous measurements of motive variants and it considers multiple criteria of effective leadership simultaneously. This allows us to examine if general theoretical propositions about the role of motive variants for leadership (i.e., that a power motive is desirable whereas an affiliation motive is not^23^) apply to the *explicit* motivational system. The present research finds no evidence that these propositions apply with regard to cooperation or with regard to ratings in a situation requiring cooperation (i.e., in the laboratory study). Hopefully, the introduction of *explicit* measures of motive variants will promote the accumulation of empirical findings that can be aggregated more easily than findings from previous studies using implicit measures (cf. Columns 11–13 in Supplementary Table S1). In future research, it may also be interesting to examine how variants of affiliation and power motives relate to other criteria of effective leadership such as entrepreneurial success^76^ which have not been considered in this study. Furthermore, it is possible that situational factors (such as crises or cultural values) and follower characteristics (such as parenting or empathy) moderate the effectiveness of motives in leaders^105,106^.

### Motives and evaluations of leaders

This work emphasizes how little approval cooperators gain. Approval from peers influences what activities people enjoy and, in turn, what behaviour they perpetuate in the future^107^. By examining the link between cooperation and leadership evaluations, this study draws attention to a critical pattern of evaluation—the tendency to approve of antisocial forms of influence such as causing oil spills. This is consistent with previous research which found that overconfidence^108^ and norm violations^109^ signal status to others.

Being perceived as assuming a leadership role appears to be a central mechanism underlying positive evaluations of selfish behaviour. Understanding this mechanism offers two potential targets for interventions: first, the link between selfishness and perceived leadership emergence and second, the link between perceived leadership emergence and transformational leadership. Scholars have questioned if leadership emergence is always aligned with the effectiveness of a team or an organization^110^. Leadership emergence—i.e., becoming influential in the eyes of others^111^—should not be equated with effective leadership^112^, and even less so in the short term^113–115^. Future research may develop interventions that base on these findings in order to weaken the link between leadership emergence and ratings of leader effectiveness, potentially reducing negative consequences from so called over-emergence^116^ due to selfish behaviour.

Selfish behaviours such as causing oil spills are in line with masculine leadership stereotypes^97,100^ and are enacted more often by men in this study. Given that mere awareness of gender-based discrimination does not preclude raters from patterns of evaluation that are in line with masculine leadership stereotypes^97,100^, we call for the development of specific interventions that help individuals reflect on their evaluation of selfish vs. cooperative behaviour in leaders. We also suggest that organizations lead by example and publicly convey their appreciation of cooperative leaders and their disapproval of selfishness^117^. Such messages are expected to attract cooperative individuals into leadership positions^118–121^, which has been found to increase overall levels of cooperation^102^ and may reduce discrimination against leaders who do not fit a masculine leadership stereotype^91,92,122^. While previous research has suggested to foster a *general* power motive among women^123^, we see no indication in our data to prioritize such an approach (given that men reported a stronger *dysfunctional* power motive whereas both genders reported similar levels on the *functional* power motive). Finally, we do not wish to say that cooperative individuals will necessarily be the most successful leaders in all circumstances. It may be possible that cooperative leaders generate short-term costs with regard to, for instance, negotiation outcomes^124^ or firm innovation^125^.

### Strengths and weaknesses

This study has some strengths and weaknesses. In the laboratory study, we assessed two behavioural measures as our outcome variables (verbal encouragement of cooperation, number of oil spills caused). This was in response to calls for research that applies multiple methods and includes behavioural measures^126,127^. Both outcomes did not rely on self-report and occurred at critical junctures during the interaction in groups^128^ (Supplementary Fig. S3, Supplementary Information, Section 4). At the same time, these measures are likely to contain a substantial amount of error variance. In line with this criticism, we only find a small and non-significant relationship between the dysfunctional power motive and encouragement of cooperation (*β* = −0.14, *P* = 0.054). The other hypothesized relationships with these behavioural outcomes are also only modest in size (*β*s = |0.23| to |0.25|, *Ps* < 0.0014) indicating that large amounts of variance in these outcomes remain unexplained.

Another critical aspect of our study is our approach to measuring functional and dysfunctional variants of affiliation and power motives. We created short self-report scales because we were not aware of any other way that would have allowed us to measure the affiliation motive as it was conceptualized in previous research (i.e., to measure the *dysfunctional* variant as conceptualized in ref.^23^ or to measure the *functional* counterpart to the dysfunctional affiliation motive (which might help explain inconsistent findings involving the affiliation motive^28,36,52–61^). Existing scales for the power motive^37,78,79^ also did not match the original^23,25,26^ conceptualization of the two faces of the power motive. On the one hand, creating new scales might be perceived as a strength of this study because these scales allowed us to test propositions involving specific motive variants. These scales are now available to be used and improved by other researchers. On the other hand, these scales have suboptimal psychometric properties which limit their value. Most importantly, each scale consists of only 4 items which may have contributed to their relatively low reliability (0.52 < *α* < 0.75 in the field survey; 0.54 < ICC < 0.87 in a pilot study with monthly measurements over a quarter year). In addition, some items have considerable cross-loadings on one or more other motive variants (see Supplementary Table S9). We tested various psychometric characteristics of these scales including unidimensionality, discriminant validity, and their factorial structure which are described in Supplementary Information, Section 2. At best, an *α* coefficient of 0.53 (for the functional affiliation motive) sets an upper limit for that scale’s validity to 0.73 (i.e., to the square-root of reliability) and leads to underestimated effect sizes. At worst, low reliability raises problems concerning the interpretation of a measure. These scales represent a first step in developing measures for functional and dysfunctional variants of affiliation and power motives. We suggest that future research builds upon these scales and creates much longer versions of them with improved psychometric characteristics.

Among the strengths of the present work are the combination of data from laboratory and field assessments (including actual leaders), leadership ratings from different sources (group members, peers), a total number of 1,161 participants (not counting the 739 peers), and the inclusion of a number of relevant control variables (implicit motives, reasoning ability, motivation to lead, fairness, and other personality characteristics).

## Conclusion

We conclude that the functional affiliation motive seems to be desirable in leaders. However, those who are high in this motive (among many are women) are not always appreciated for that. Raters sometimes interpret selfish acts as leadership behaviour. Those who are aware of gender-based discrimination are not immune to this pattern of evaluation (but even appear to be at increased risk).

## Materials and Methods

### Participants

#### Laboratory study (Settlers of Catan)

For sample size, we set a target of “200” before we started collecting data. Participants are 201 individuals (103 women) aged *M* = 24 y (s.d. = 6). Most are students (89%) majoring in psychology (51%). Some presently hold or formerly (in their last employment) held a professional leadership position (17%). We recruited participants on campus and through local advertisements. All participants received a variable payment of *M* = €6.94, s.d. = 1.89, in addition to either a fixed amount of €20 (available to all participants except psychology students) or course credit (available to psychology students) for a total duration of approx. 4 h (with an additional €2 or course credit for every 15 min beyond 4 h 10 min). Fresh organic fruits, snacks, as well as hot and cold beverages were available to participants free of charge.

#### Field survey

Budget (€2,000 for the final wave of recruitment) determined sample size. Respondents were 961 individuals (513 women) aged *M* = 31 y (s.d. = 12). Most of them have work experience (73%) of, on average, 9 y (s.d. = 12). Some presently hold or formerly held (if not working anymore) a professional leadership position (27%). We recruited half of them via an online labour market and the other half through local advertisements and social networks. Respondents received approx. €2.50 for 15–25 min. Respondents recruited 739 peers (439 women) who are either friends/acquaintances (43%) and family/partners (43%) of the respondents, or work together with respondents (14%). In total, we obtained one or more peer ratings for 486 of the respondents. Peers were not compensated.

### Procedure of the laboratory study (Settlers of Catan)

We distributed data collection over two occasions *M* = 19 days (s.d. = 30) apart from each other. At Time 1, we measured all independent variables in an online survey. At Time 2, participants came to the laboratory and interacted with other participants. We informed participants in the beginning of both occasions that they were going to be videotaped at Time 2. All participants provided informed consent online (Time 1) and with their signature (Time 2). We explicitly notified participants before we started recording video. Both cameras and two video lights were clearly visible.

After completing the survey at Time 1, participants automatically received regular emails with personalized invitations for Time 2 through a custom-coded script, until they registered for a particular date. Personalizing invitations in this way allowed us to stratify group composition. We intended that all groups contain 2 male and 2 female individuals. If multiple group members were psychology students, they were not allowed to belong to the same cohort so that most group members would not know each other. This procedure resulted in *n* = 45 complete groups with 4 members each (2 male, 2 female) and *n* = 7 smaller groups with 3 members each in case that one person did not show up. We control for group size in all analyses. The average degree of familiarity between group members was *M* = 1.2 (s.d. = 0.6) on a scale of 1 to 6.

In the laboratory, at Time 2, participants first had a group discussion about the solution to a fictitious rescue scenario (i.e., *The Desert Dilemma*^129^) which lasted up to 15 min (*M* = 11:00 min, s.d. = 2:55). Group members could thereby get to know each other. After the discussion, group members rated each other for the first time on transformational leadership and on assuming a leadership role. They provided these ratings on individual workstations separated by dividing walls. However, they could see the backs of all other group members and tags with their first names on it which were attached at various positions. We use these ratings as a baseline measure in all analyses involving transformational leadership and perceived influence, respectively. A short break followed.

After the break, we instructed participants for a second time (the first time was at the end of the online part of this study) about the rules of the Settlers of Catan game and, in particular, about the Oil Springs iteration of this game^96^. We handed over all different pieces of the game to each participant so that they could familiarize themselves with them by themselves at their workstation before sitting down with the others at a table in the center of the room with the game on it. The experimenter assured participants that they could ask about the rules of the game at any time. All questions were answered at all times as long as they were related to the understanding of the game.

In the Settlers of Catan game, all players manage their own population. The goal is to grow one’s population on an island that all players share. Players earn so called *victory points* for constructing buildings, long roads, or for sequestering (instead of using) oil. To be able to build anything, players need resources which they obtain over time or by trading with other players. We chose the Oil Springs iteration of this game, which simulates the real-world issues associated with global consumption of fossil fuels^96^. The Oil Springs scenario allows players to drill for oil and utilize it to grow their populations faster. All use of oil is indicated on the board so that all players are aware of it. After each fifth oil that is being used by any one of the players, an oil spill happens. Such a disaster either destroys one of the perimeters of the island and its future capacity to produce resources (approx. 80% likelihood) or causes coastal flooding which destroys all settlements located directly on coasts (approx. 20% likelihood). This creates a social dilemma of the type of a resource dilemma. While a single player benefits from using oil, the whole group suffers from deterioration of future productivity as a result of that player’s oil use. The game was over after 10 rounds (40 moves in groups of 4 and 30 moves in groups of 3, *M* = 76 min, s.d. = 26). However, we concealed this fact from participants. Not knowing how long the game would last made it impossible for participants to anticipate the extent of future losses of productivity due to oil spills (see Supplementary Information, Section 4 for a discussion of the utility of oil use). Participants received financial incentives based on the results of the game. These incentives were intended to activate motives related to both cooperation and selfishness. Participants knew that after the game, a coin toss determined whether they received a payment based on group performance (intended to activate motives related to cooperation) or on individual performance (intended to activate motives related to selfishness). Previous research has found that activation of motives increases their impact on behaviour^46^. The ambiguity that is introduced by coupling the payment to a coin toss resembles the ambiguity that occurs in real-life social dilemmas. In reality, there is often uncertainty involved whether cooperation pays off. Performance is indicated by the number of victory points a player earns during the game. All victory points exceeding a cutoff of 5 were worth €1 per point (*M* = €1.13, s.d. = 1.47). Supplementary Information, Section 3 describes further modifications that we made to the original procedure of the game.

After the game, all players rated each other again on transformational leadership and on assuming a leadership role. Finally, we asked participants whether they would recommend the study to others, to which 99% answered “yes”. After completing all questionnaires, we compensated participants and thanked them for their contribution. If they had any questions about the study, we tried to answer them as well as we could. We only requested that they would not share any strategies or ideas with their friends, if those friends might want to participate in the study. All procedures were in line with all relevant ethical regulations described in the Ethics Code of the American Psychological Association. The Technische Universität Darmstadt institutional review board provided guidelines for study procedures. All procedures were approved by the University of Bamberg institutional review board.

### Procedure of the field survey

The survey was conducted online. All scales were presented in randomized order. We used 6-point scales if not otherwise indicated. All participants provided informed consent. All scales were answered by the respondents using self-report measures except for the peer ratings of general leadership competence for which respondents’ peers rated respondents’ general leadership competence. All procedures were approved by the Technische Universität Darmstadt institutional review board.

### Measures used in both studies

#### Motives

We define the *functional affiliation motive* as a desire for social interactions that are sincere and considerate, fostering deep and honest relationships with others. A sample item is “I wish that people like me for being sympathetic and cooperative”. We define the *dysfunctional power motive* as a drive for possessing and using authority in order to serve one’s personal interests. A sample item is “it pleases me to have a lot of power and influence, because there are many people that you need to keep under control”. We define the *dysfunctional affiliation motive* as a striving for harmonious relationships with others that is characterized by confirmation seeking and self-effacement. A sample item is “it is very important to me to be accepted by others. Therefore I sometimes say things of which I am not convinced that they are right, but that make me look good”. We define the *functional power motive* as a desire for using responsible and benevolent channels of influence. A sample item is “I enjoy to contribute something through my channels of influence that is aligned with the greater good”. Supplementary Information, Section 1 provides details on item development and item selection. Supplementary Information, Section 2 describes our findings when evaluating the newly developed scales psychometrically. Supplementary Table S7 lists the final selection of items used in this work.

#### Control variables

We measured affective *motivation to lead* with 9 items^130^. We used 6-point scales in the laboratory study and for the first *n* = 203 participants in the field survey (to keep response scales consistent across measures) but changed to the original 5-point format for the next 758 participants in the field survey (to be able to provide unpaid respondents with norm-based feedback on their motivation to lead as an incentive for participation). We assessed *personality* using a short version of the Big Five Inventory with a total of 10 items^131^ as well as the 3-item fairness facet of the Honesty-Humility factor^132^. Reliabilities, descriptive statistics, and intercorrelations of all variables used in each study are available in Supplementary Datasets 1 and 2 as well as at https://osf.io/yt4qh/. The datasets analyzed during the current study are available in the Open Science Framework repository, https://osf.io/yt4qh/, except for information that could compromise research participant privacy.

#### Demographic information

We asked participants whether they currently hold a professional leadership position, or, if they are not working at the moment (e.g., because they are retired), whether they held a leadership position at some point in the past.

### Measures used in the laboratory study (Settlers of Catan)

#### Verbal statements encouraging cooperation

We videotaped the whole conversation during the game. We count all statements that favor either cooperation or selfishness. This count reflects (*i*) statements about cooperative/selfish strategies (e.g., “we should share the resources that everyone needs” vs. “I think it is best if everyone does their own thing”) and (*ii*) more general statements expressing a positive/negative attitude towards the group (e.g., “great, now everyone has more than 5 victory points“ vs. “I don’t care what happens when I cause an oil spill”). We count all statements that (*i*) initiate a conversation about a topic related to cooperation, (*ii*) support such an initiative, or (*iii*) reject such an initiative (*reverse coded*, i.e., counting toward the other category). As support or rejection, we count only instances where a person makes an active statement. We do not count one word answers, nodding, or shaking one’s head.

For both statements encouraging cooperation and statements encouraging selfishness, we log-transform count values to reduce the weight of statements that are repetitions of a player’s position relative to statements that reveal a player’s position for the first time. Agreement over two trained raters is *r* = 0.79, *P* < 0.0001 for statements encouraging cooperation and *r* = 0.78, *P* < 0.0001 for statements encouraging selfishness. Next, we *z*-standardize statements encouraging cooperation (*M*_raw_ = 4.8, s.d. = 5.9) and statements encouraging selfishness (*M*_raw_ = 1.6, s.d. = 2.6) separately. Given that statements encouraging selfishness are more rare, we assume that they have a higher weight per statement in the conversation. By standardizing both types of statements separately before aggregating them, we assign an equal weight to both indices. When aggregating both indices, we assign a negative sign to statements encouraging selfishness. Inter-rater agreement is *r* = 0.73, *P* < 0.0001. Finally, we aggregate the resulting aggregates from both raters. Without log transformation in the beginning, the final aggregates would have had higher kurtosis (9.34 vs. 0.95, s.e.m. = 0.34). All count values are available on https://osf.io/yt4qh/.

#### Oil spills

During the game, the experimenter noted all moves on a custom-made form (available at https://osf.io/yt4qh/). Any inconsistencies in the record (the occurrence of an oil spill was logged at two different places) were resolved by replaying the game on video.

#### Ratings

Participants rated each other immediately after the game of Settlers of Catan “with respect to the behaviour of [each group member, referred to by their first names] during the whole experiment”. To ensure that all ratings are only based on behaviour during the game (instead of being based on non-behavioural information^133^), we control for baseline ratings that we measured in the beginning of the experiment after a short group discussion. There, we asked respondents to provide all answers “with respect to the behaviour of [each group member, referred to by their first names] during the group discussion”.

*Transformational leadership* describes the extent to which followers feel inspired and supported by a leader to work towards common goals. We assessed transformational leadership with a German language adaptation^134^ of the Multifactor Leadership Questionnaire^135^. This adaptation measures 6 facets of transformational leadership. For each facet, we chose the item with the highest loading on one of the two factors representing transformational leadership^134^, resulting in a total of 6 items answered on 5-point scales.

We measured *assumed leadership role* with 2 items answered on 5-point scales reading (*i*) “to what extent has [first name] shown leadership behaviour?” and (*ii*) “[during the group discussion/the whole experiment], [first name] has assumed a leadership role”.

#### Awareness of gender-based discrimination

We measured this variable with 3 items from the *denial of continuing discrimination* subscale of the *modern sexism scale*^136^. Of its 5 items, we chose the ones that are either general or work-related, e.g., “women often miss out on good jobs due to sexual discrimination”. We coded the scale so that high values indicate high awareness of gender-based discrimination (and, thus, low modern sexism).

#### Control variables

We measured an *implicit affiliation motive* and an *implicit power motive* using the approach of the picture story exercise. We showed respondents a picture for 10 s and then asked them to come up with a story surrounding the depicted situation within 4 min per picture. We used 3 pictures—*women in laboratory*^137^, *mad scientist*^138^, and *nightclub scene*^138^. Respondents’ stories were then coded for motive imagery by a trained coder using Winter’s coding system for running text. For example, if a character in one of the stories attempts to influence another character, that particular sentence of that particular story is coded as power imagery. Activity inhibition is coded by counting how often the word *not* is used^139^. We correct for word count using regression analysis.

We measured an *achievement motive* with 4 items from a German questionnaire (the business focused inventory of personality^140^). We chose items 22, 85, 159, and 172, because these items had the highest factor loadings of all items that are phrased general enough for our purpose, e.g., “even after a very good performance, I still seek improvement”.

We measured *reasoning ability* with the short version of the Hagen Matrices Test^141^. This version consists of 6 3 × 3 matrices with 8 response options each. Each matrix needs to be completed within 2 min.

### Measures used in the field survey

#### Selfish business decisions

Respondents read six detailed descriptions of hypothetical business scenarios^142^. All scenarios involve social dilemmas. Each decision requires balancing personal benefits against expected harm to society, the environment, or legal liability. Respondents indicated on 6-point scales how likely it was that they would make a selfish decision.

#### General leadership competence

Peers rated respondents on 3 items measuring their leadership competence in general, e.g., “the person that I am rating is/would make a good leader”. We asked respondents to nominate peers who know them very well. Peers indicated that they know respondents well, *M* = 5.5 (s.d. = 0.9) on a scale of 1 to 6. Family members (29%) gave the highest ratings (*r* = 0.15, *P* < 0.0001) whereas friends (38%, *r* = −0.09, *P* = 0.043) and acquaintances (6%, *r* = −0.13, *P* = 0.0018) gave the lowest ratings. We collapsed ratings across these different types of peers because we wanted to include all respondents regardless of external circumstances that might have influenced peer nomination (e.g., occupational status, availability of friends and family members, or partnership status).

## Supplementary information

Supplementary Information

Dataset 1 (laboratory study)

Dataset 2 (field survey)

## Data Availability

Data are available for this paper at https://osf.io/yt4qh/.
