# Supplementary material for: Motives relate to cooperation in social dilemmas but have an inconsistent association with leadership evaluation
Source: Sci Rep. 2019 Jul 12;9:10118. doi: 10.1038/s41598-019-45931-4 (PMC6626160; doi:10.1038/s41598-019-45931-4)
Supplement: Supplementary file 1 — Supplementary Information [file 41598_2019_45931_MOESM1_ESM.pdf]

Supplementary Information

Motives relate to cooperation in social dilemmas but have an inconsistent association with leadership evaluation

Christian Wolff*[1,2] & Nina Keith[2]

[1]University of Bamberg

[2]Technische Universität Darmstadt

*christian.wolff@uni-bamberg.de

Study	Year	Overlapping samples	*n*	Sector/context	Sample	Motive measure	Dependent variable(s)	Zero-order correlation		Motive combination/variants		
								Power motive	Affiliation motive	Analytical approach	Label and formula	Central finding(s)
McClelland & Boyatzis, Sample 1[42]	1982	No	92	Telecommunication	Male managers with engineering responsibilities	Picture Story Exercise (6 pictures)	*Objective data*<br>Leader career success (after 8 and 16 y)			Configuration	Leadership motive pattern: *n*Pow ≥ 45, *n*Pow ≥ *n*Aff, AI > median, AI_{RAW} ≥ 2	Leadership motive pattern not related to career success
McClelland & Boyatzis, Sample 2[42]	1982	No	144		Male managers without engineering responsibilities	Picture Story Exercise (6 pictures)	*Objective data*<br>Leader career success (after 8 and 16 y)			Configuration	Leadership motive pattern: *n*Pow ≥ 45, *n*Pow ≥ *n*Aff, AI > median, AI_{RAW} ≥ 2	Leadership motive pattern positive for career success
Cornelius & Lane, Sample 1[43]	1984	No	18	Education	Curriculum directors	Picture Story Exercise (6 pictures)	*Objective data*<br>Administrative efficiency<br>Center size<br><br>*Ratings by subordinates*<br>Employee satisfaction<br>Team spirit<br>Organizational clarity			Difference values	Leadership motive pattern: *n*Pow – *n*Aff	 –0.42*<br>0.59**<br><br><br>–0.36†<br>–0.20<br>–0.18
Cornelius & Lane, Sample 2[43]	1984	No	21		Center managers	Picture Story Exercise (6 pictures)	*Objective data*<br>Administrative efficiency<br>Center size<br><br>*Ratings by subordinates*<br>Employee satisfaction<br>Team spirit<br>Organizational clarity			Difference values	Leadership motive pattern: *n*Pow – *n*Aff	 –0.09<br>0.23<br><br><br>0.04<br>–0.26<br>–0.21
Sorrentino & Field[44]	1986	No	48	Laboratory (5 sessions, 1.5 to 2 h/ session, groups of 4)	Male psychology students	Descriptive sentences were used to elicit stories (4 sentences)	*Ratings by group members*<br>Task leadership<br>Socioemotional leadership<br>Leadership emergence 1st choice<br>Leadership emergence 1st & 2nd c.		 *P* = 0.015<br>*P* = 0.042<br>*P* = 0.04<br>*P* = 0.0005<br>(all effects positive)	Interactions via dichotomized variables	Two-way interaction between *n*Aff and *n*Ach	Interaction between *n*Aff and *n*Ach positive for 2 of 5 outcomes

Winter[45]	1987	No	14-31	Government/ politics	US presidents	Speech (first inaugural address)	*Objective data/coded variables*<br>Vote percentage<br>Margin of victory<br>Reelected<br>Reelected (all instances)<br>% vote for party's House candidates<br>Court/cabinet rejections<br>Percentage vetoes overridden<br>Adjusted midterm House loss<br>War entry<br>War avoidance<br>Arms limitation<br>Consensus of greatness<br>Great decisions cited	 –0.04<br>–0.07<br>0.06<br>0.27<br>0.13<br>–0.19<br>0.01<br>–0.23<br>0.52^{**}<br>$0.34^{\dagger}$<br>–0.05<br>0.40^{*}<br>0.51^{**}		Difference values	*n*Pow – *n*Aff	 0.10<br>0.05<br>–0.05<br>0.16<br>0.20<br>–0.20<br>–0.04<br>0.03<br>$0.36^{\dagger}$<br>0.16<br>$–0.55^{*}$<br>0.35<br>0.27
Spangler & House[46]	1991	Yes (Winter, 1987[45])	29-39	Government/politics	US presidents	Speech (first inaugural address)	*Objective data/coded variables*<br>War entry<br>War avoidance<br>Consensus of greatness<br>Great decisions<br>Mean greatness<br>Social performance<br>Economic performance<br>International relations performance	 0.52^{**}<br>0.33<br>0.40^{*}<br>0.51^{**}<br>0.26<br>0.17<br>0.00<br>0.27	 0.17<br>0.23<br>0.09<br>0.30<br>–0.18<br>–0.20<br>0.07<br>0.00	Configuration and interactions (both multiplicative and dichotomized) in the same multiple regression model	Syndrome: *n*Pow ≥ 45, *n*Pow ≥ *n*Aff, AI ≥ median; two-way interaction between *n*Pow and AI	Syndrome and interaction positive for 0 of 5 outcomes
House et al.[47]	1991	Yes (Winter, 1987[45])	31	Government/politics	US presidents	Speech (first inaugural address)	*Objective data/coded variables*<br>Charisma<br>Direct action<br>Subjective performance<br>International relations performance<br>Economic performance<br>Social performance			Multiple regression analysis	Simultaneous main effects of *n*Pow, *n*Aff, *n*Ach, AI, charisma, crises, and age	Power positive for 5 of 6 outcomes
Winter[48]	1991	Yes (McClelland & Boyatzis, 1982[42])	141	Telecommunication	Male managers without engineering responsibilities	Picture Story Exercise (6 pictures)	*Objective data*<br>Leader career success (after 16 y)			Direct coding of responsibility; configural approach	Responsible power: *n*Pow ≥ 50, responsibility ≥ 45; responsible power motivation pattern: *n*Pow > 45, *n*Pow ≥ *n*Aff, responsibility ≥ 45	Responsible power and responsible power motivation pattern positive for career success

Winter[49]	1993	No	58	Government/politics	British go-<br>vernment	Sovereign's<br>speech/<br>speech from<br>the throne	*Objective data/coded variables*<br>War entry (1 year prior)<br>War entry (2 years prior)<br>War entry (3 years prior)			Difference<br>values	*n*Pow – *n*Aff	0.30*<br>0.21<br>–0.05
Jacobs & McClelland, Sample 1[50]	1994	No	229	Telecommunication	Entry-level managers who stayed in the company	Picture Story Exercise (2 pictures)	*Objective data*<br>Leader career success (after 12 y)	*ns*	*ns*	Direct coding of power themes; configural approach	Standard leadership motive pattern: *n*Pow ≥ 45, *n*Pow ≥ *n*Aff, AI ≥ median; modified leadership motive pattern: *n*Pow ≥ 45, *n*Pow ≥ *n*Aff; power themes: resourceful vs. reactive vs. helpless power	No significant relationships for motive patterns or variants
Jacobs & McClelland, Sample 2[50]	1994	No	56	Telecommunication	Entry-level managers who left the company	Picture Story Exercise (5 pictures)	*Objective data*<br>Leader career success (after 12 y)	0.34**	–0.30*	Direct coding of power themes; configural approach	Standard leadership motive pattern: *n*Pow ≥ 45, *n*Pow ≥ *n*Aff, AI ≥ median; modified leadership motive pattern: *n*Pow ≥ 45, *n*Pow ≥ *n*Aff; power themes: resourceful vs. reactive vs. helpless power	Modified leadership motive pattern positive for career success; successful male managers used reactive power themes while successful female managers used resourceful power themes
Langner & Winter, Sample 1[51]	2001	No	67	Political crises	Documents by government officials	Official public statements and letters	*Objective data*<br>Net concessions during political crises	–0.22†	0.23†			
Kirkpatrick et al., Sample 1[52]	2002	No	269	Architectural woodworking	Entrepreneurs in the role of the CEO	Vision statements	*Objective data*<br>Venture growth (sales, employment, and profit over a period of 2 y, controlling for previous growth)	0.11*	0.07			

Kirkpatrick et al., Sample 2[52]	2002	No	82	Federal engineering services	Supervisory managers	Vision statements	*Ratings by subordinates*<br>Manager performance<br><br>*Ratings by manager*<br>Unit performance	−0.06<br><br><br>−0.16†	0.19*<br><br><br>0.21*			
Schultheiss & Brunstein[53]	2002	No	68	Mixed	Students and employees	Picture Story Exercise (6 pictures)	*Ratings by observers*<br>Persuasiveness	−0.01		Interactions in multiple regression analysis	Inhibited power motive: two-way interaction between *n*Pow and AI	Inhibited power motive positive for persuasiveness
De Hoogh et al.[54]	2005	No	73	Wide range of for profit and non-profit organizations	CEOs	Semistructured interviews about the CEOs' role and their functioning as a manager (45 to 60 min)	*Ratings by subordinates*<br>Charismatic leadership<br>Organizational commitment	0.28*<br>−0.10	−0.18<br>−0.10	Interactions in hierarchical regression analysis	Two-way interaction between *n*Pow and reponsibility and three-way interaction between *n*Pow, responsibility, and organization type	Irresponsible power positive for charismatic leadership in for profit organizations
Magee & Langner, Sample 1[6]	2008	No	90	Laboratory experiment (political conflict)	Students and university employees	Participants' drafts of a letter to Premier N. S. Krushchev on behalf of US President J. F. Kennedy	*Self-reported decisions*<br>Advised escalation of conflict		0.00	Direct coding of motive variants (Winter, 1973[60])	Personalized power: hope for power; socialized power: fear of power (Winter, 1973[60])	Personalized power positive for escalation of conflict
Magee & Langner, Sample 2[6]	2008	No	69	Laboratory experiment (healthcare)	Students and university employees	Fifteen written personal strivings in participants' everyday lives	*Self-reported decisions*<br>Recommendation for approving a beneficial (but risky) drug		0.04	Direct coding of motive variants (Winter, 1973[60])	Personalized power: hope for power; socialized power: fear of power (Winter, 1973[60])	Socialized power positive for drug approval

Kazén & Kuhl[55]	2011	No	382	Mixed	Executive managers	Operant Motive Test (15 pictures)	*Self-reported well-being*<br>Well-being<br>Stress	 0.01<br>–0.03				
Delbecq et al.[56]	2013	No	28	Technology (Silicon Valley)	CEOs	Semistructured interviews about concerns, beliefs, values, opinions, and management philosophies (45 to 60 min)	*Ratings by subordinates*<br>Participative leadership<br>Instrumental leadership<br>Charismatic leadership<br>Follower motivation<br>Exceptional performance<br>Teamwork	 0.08<br>–0.25<br>–0.17<br>–0.24<br>–0.16<br>–0.04	 0.13<br>0.22<br>0.28<br>0.27<br>0.12<br>0.12			
Howard, Sample 1[57]	2013	Yes (McClelland & Boyatzis, 1982[42])	101	Telecommunication	Male managers with engineering responsibilities	Picture Story Exercise (4 to 6 pictures)	*Objective data*<br>Leader career success (after 25 y)<br>Leader career success (after 25 y), predictors measured at year 8	 0.08<br>–0.04	 –0.09<br>0.06	Configuration	Leadership motive pattern: *n*Pow ≥ 45, *n*Pow ≥ *n*Aff, AI ≥ median; leader motive without AI: *n*Pow ≥ 45, *n*Pow ≥ *n*Aff	Both leadership motive patterns not related to career success at year 25
Howard, Sample 2[57]	2013	Yes (McClelland & Boyatzis, 1982[42])	174	Telecommunication	Male managers without engineering responsibilities	Picture Story Exercise (4 to 6 pictures)	*Objective data*<br>Leader career success (after 25 y)<br>Leader career success (after 25 y), predictors measured at year 8	 0.03<br>0.01	 –0.25**<br>0.03	Configuration	Leadership motive pattern: *n*Pow ≥ 45, *n*Pow ≥ *n*Aff, AI ≥ median; leader motive without AI: *n*Pow ≥ 45, *n*Pow ≥ *n*Aff	Both leadership motive patterns not related to career success at year 25
Howard, Sample 3[57]	2013	Yes (Jacobs & McClelland, 1994[50])	111	Telecommunication	Managers who left the organization	Picture Story Exercise (4 to 6 pictures)	*Ratings by manager*<br>Leader career success (after 25 y)<br>Salary (after 25 y)	 0.00<br>–0.01	 0.04<br>–0.03	Configuration	Leadership motive pattern: *n*Pow ≥ 45, *n*Pow ≥ *n*Aff, AI ≥ median; leader motive without AI: *n*Pow ≥ 45, *n*Pow ≥ *n*Aff	Both leadership motive patterns not related to career success and salary at year 25

Steinmann et al.[58]	2015	No	70	Mixed	Managers	Picture Story Exercise (6 pictures, 5 min/ picture)	*Ratings by manager*<br>Goal attainment of the team<br>Developments in income	 –0.11<br>0.06	 0.09<br>–0.11	Interactions in hierarchical regression analysis	Compassionate leadership profile: two-way interactions between *n*Pow, *n*Aff, and AI and three-way interaction between *n*Pow, *n*Aff, and AI	Three-way interaction between *n*Pow, *n*Aff, and AI positive for 2 of 2 outcomes
Steinmann et al.[59]	2016	No	70	Mixed	Managers	Picture Story Exercise (6 pictures, 4 min/ picture)	*Ratings by subordinates*<br>Transformational leadership<br>Passive leadership<br>Concern for followers' needs<br>Job satisfaction<br>Satisfaction with the leader<br>In-role performance<br>Organizational citizenship behavior<br><br>*Ratings by manager*<br>Developments in income	 0.09<br>0.03<br>0.11<br>0.01<br>0.09<br>–0.03<br>–0.18<br><br><br>–0.11	 0.14<br>–0.04<br>0.25*<br>0.14<br>0.06<br>0.12<br>0.15<br><br><br>–0.18	Interactions in hierarchical regression analysis	Two-way interactions between *n*Pow, *n*Aff, *n*Ach, and AI and three-way interaction between *n*Pow, *n*Aff, and *n*Ach	Three-way interaction between *n*Pow, *n*Aff, and *n*Ach positive for 4 of 6 outcomes

Table S1. Previous studies on the relationship of implicit power and affiliation motives, their combinations, and/or their variants with leadership and leader outcomes (k = 26, n = 2,495 participants). Notes: We excluded studies that (*i*) did not provide enough information (McClelland & Burnham, 1976[1]; Burnham, 1997[61]; Lukié, 2015[62]; Winter, 2018[63]), (*ii*) were not specific for leadership (Jenkins, 1994[64]; Winter et al., 1998[65]), or (*iii*) were unavailable to us (Winter, 1979[66]). CEO = chief executive officer, *n*Pow = need for power/ power motive, *n*Aff = need for affiliation/affiliation motive, *n*Ach = need for achievement/achievement motive, and AI = activity inhibition. This overview only includes information about the achievement motive if it was central to a study that also included the power and/or affiliation motive. The lists of dependent variables are not exhaustive. ** $P < 0.01$, * $P < 0.05$, † $P < 0.10$.

Predictor	Leaders (n = 257)			Workers (n = 446)			Students (n = 258)		
	β	***t***	***P***	***β***	***t***	***P***	***β***	***t***	***P***
Functional affiliation motive	–0.18	–3.10	0.002	–0.19	–4.04	0.000	–0.25	–3.86	0.000
Dysfunctional affiliation motive	–0.08	–1.34	0.182	0.10	2.07	0.039	0.03	0.44	0.661
Functional power motive	–0.10	–1.62	0.107	–0.07	–1.42	0.157	0.09	1.25	0.212
Dysfunctional power motive	0.60	11.03	0.000	0.38	8.27	0.000	0.35	5.36	0.000

Table S2. Motives relate to selfish business decisions across different occupational statuses. Notes: *Leaders* state that they currently hold a professional leadership position or, if they are not working anymore, held one in the past. *Workers* report having work experience (but no leadership position). *Students* are either students or homemakers.

Predictor	Encouragement of cooperation ($n = 201$)			Oil spills ($n = 201$)			Selfish business decisions ($n = 960$)		
	β	***t***	***P***	***β***	***t***	***P***	***β***	***t***	***P***
Simple model									
Group size (3 vs. 4)	–0.03	–0.50	0.620	0.16	2.37	0.019			
Functional affiliation motive	0.25	3.37	0.001	–0.25	–3.38	0.001	–0.20	–6.30	0.000
Dysfunctional affiliation motive	–0.00	–0.06	0.955	–0.01	–0.09	0.927	0.03	1.03	0.301
Functional power motive	0.09	1.08	0.283	0.01	0.17	0.863	–0.06	–1.60	0.111
Dysfunctional power motive	–0.14	–1.94	0.054	0.23	3.26	0.001	0.44	14.32	0.000
Extended model									
Group size (3 vs. 4)	–0.02	–0.22	0.824	0.13	1.89	0.060			
Neuroticism	0.11	1.43	0.154	–0.02	–0.30	0.767	–0.10	–3.33	0.001
Extraversion	0.08	0.93	0.354	0.11	1.46	0.147	0.01	0.22	0.830
Openness	0.10	1.45	0.150	–0.05	–0.71	0.478	–0.13	–4.89	0.000
Agreeableness	–0.06	–0.75	0.455	0.00	0.06	0.955	–0.06	–2.06	0.039
Conscientiousness	–0.04	–0.43	0.665	–0.19	–2.32	0.022	–0.01	–0.31	0.756
Fairness	0.14	1.83	0.068	–0.13	–1.67	0.097	–0.31	–10.67	0.000
Reasoning ability	–0.04	–0.54	0.588	0.03	0.49	0.627			
Achievement motive	0.10	1.19	0.236	–0.05	–0.56	0.578			
Motivation to lead	–0.03	–0.31	0.760	–0.03	–0.34	0.735	–0.02	–0.63	0.526
Implicit affiliation motive	–0.08	–1.09	0.277	–0.03	–0.36	0.720			
Implicit power motive	–0.04	–0.50	0.620	0.11	1.57	0.118			
Activity inhibition	0.13	1.77	0.078	0.00	0.02	0.984			
Affiliation × power	–0.08	–1.07	0.289	–0.04	–0.52	0.603			
Affiliation × activity inhibition	–0.00	–0.04	0.969	0.02	0.30	0.765			
Power × activity inhibition	0.09	1.19	0.237	–0.04	–0.59	0.556			
Affiliation × power × activity inhibition	–0.16	–1.87	0.063	–0.05	–0.54	0.588			
Functional affiliation motive	0.23	2.74	0.007	–0.18	–2.22	0.028	–0.12	–3.73	0.000
Dysfunctional affiliation motive	–0.03	–0.32	0.751	–0.04	–0.40	0.688	0.05	1.42	0.156
Functional power motive	0.10	1.03	0.306	0.02	0.17	0.865	–0.03	–0.86	0.389
Dysfunctional power motive	–0.11	–1.18	0.241	0.13	1.40	0.164	0.33	10.00	0.000

Table S3. Motives relate to cooperation in social dilemmas. Notes: *Group size* is coded so that 3 persons = 3 ($n = 7$) and 4 persons = 4 ($n = 45$).

Predictor	Leadership ratings (after social dilemma, *n* = 201)			Leadership ratings (in general, *n* = 486)			Leadership position (*n* = 961)		
	β	***t***	***P***	***β***	***t***	***P***	***β***	***t***	***P***
Simple model									
Baseline values	0.70	14.02	0.000						
Group size (3 vs. 4)	0.10	1.94	0.054						
Functional affiliation motive	0.17	3.08	0.002	0.03	0.73	0.467	–0.06	–1.79	0.074
Dysfunctional affiliation motive	–0.09	–1.57	0.118	–0.09	–1.80	0.072	–0.08	–2.10	0.036
Functional power motive	0.02	0.26	0.799	0.25	4.80	0.000	0.21	5.44	0.000
Dysfunctional power motive	–0.06	–1.08	0.283	0.03	0.68	0.498	–0.03	–0.84	0.404
Extended model									
Baseline values	0.68	12.74	0.000						
Group size (3 vs. 4)	0.08	1.57	0.118						
Neuroticism	–0.02	–0.31	0.755	0.04	0.90	0.369	–0.05	–1.33	0.183
Extraversion	0.02	0.37	0.715	0.08	1.64	0.102	–0.02	–0.65	0.513
Openness	–0.01	–0.21	0.834	–0.07	–1.62	0.106	0.04	1.24	0.216
Agreeableness	0.08	1.32	0.190	0.00	0.03	0.980	0.08	2.25	0.025
Conscientiousness	–0.12	–1.84	0.068	0.14	3.05	0.002	0.12	3.45	0.001
Fairness	0.04	0.69	0.494	0.06	1.32	0.187	–0.02	–0.57	0.567
Reasoning ability	0.08	1.46	0.147						
Achievement motive	0.06	0.96	0.336						
Motivation to lead	0.08	1.07	0.285	0.16	2.79	0.005	0.26	6.28	0.000
Implicit affiliation motive	–0.01	–0.20	0.841						
Implicit power motive	–0.00	–0.08	0.939						
Activity inhibition	0.04	0.76	0.447						
Affiliation × power	0.07	1.22	0.226						
Affiliation × activity inhibition	–0.05	–0.89	0.375						
Power × activity inhibition	0.03	0.50	0.620						
Affiliation × power × activity inhibition	–0.01	–0.19	0.848						
Functional affiliation motive	0.14	2.30	0.022	0.02	0.32	0.748	–0.09	–2.34	0.019
Dysfunctional affiliation motive	–0.10	–1.45	0.150	–0.04	–0.70	0.484	–0.02	–0.41	0.679
Functional power motive	–0.04	–0.63	0.533	0.16	2.83	0.005	0.09	2.09	0.037
Dysfunctional power motive	–0.05	–0.72	0.471	0.02	0.29	0.772	–0.08	–2.10	0.036

Table S4. Motives relate to leadership evaluation. Notes: *Group size* is coded so that 3 persons = 3 (*n* = 7) and 4 persons = 4 (*n* = 45). *Leadership ratings (after social dilemma)* are made on a scale measuring transformational leadership. We control for baseline values from before the game so that ratings are only based on behaviour during the game. For *leadership position*, we calculated standard multiple regression analysis to facilitate comparison of results. Binomial logistic regression analysis yields virtually identical results.

Dependent variable	Sample	Men *n*	Men Mean	Men s.d.	Women *n*	Women Mean	Women s.d.	*d*	*t*	*P*
Motive										
Functional affiliation motive	Laboratory study (Settlers of Catan)	98	4.33	0.66	103	4.73	0.57	0.64	4.56	0.000
	Field survey	448	4.24	0.71	513	4.50	0.61	0.39	5.96	0.000
	Subgroup: leaders	132	4.28	0.76	125	4.51	0.65	0.32	2.68	0.008
	Subgroup: workers	189	4.19	0.66	257	4.49	0.60	0.48	5.05	0.000
	Subgroup: students	127	4.29	0.72	131	4.51	0.60	0.33	2.58	0.010
Dysfunctional power motive	Laboratory study (Settlers of Catan)	98	3.10	0.96	103	2.65	0.90	–0.49	–3.48	0.001
	Field survey	448	3.23	0.92	513	2.93	0.95	–0.32	–4.93	0.000
	Subgroup: leaders	132	3.16	0.95	125	2.95	1.02	–0.21	–1.67	0.097
	Subgroup: workers	189	3.19	0.91	257	2.94	0.95	–0.27	–2.80	0.005
	Subgroup: students	127	3.36	0.89	131	2.90	0.88	–0.52	–4.23	0.000
Cooperation in social dilemmas										
Encouragement of cooperation	Laboratory study (Settlers of Catan)	98	–0.20	1.12	103	0.19	0.84	0.40	2.80	0.006
Statements pro cooperation		98	5.36	6.99	103	4.22	4.54	–0.20	–1.37	0.173
Statements pro selfishness		98	2.26	2.91	103	1.05	2.06	–0.48	–3.38	0.001
Oil spills		98	0.40	0.68	103	0.06	0.24	–0.67	–4.66	0.000
Selfish business decisions	Field survey	448	2.64	1.04	512	2.18	0.91	–0.47	–7.22	0.000
Leadership evaluation										
Leadership ratings (after social dilemma)	Laboratory study (Settlers of Catan)	98	–0.08	0.39	103	–0.10	0.36	–0.06	–0.43	0.667
Leadership ratings (in general)	Field survey	191	4.51	1.01	295	4.69	0.93	0.19	2.00	0.046
Leadership position		448	0.29	0.46	513	0.24	0.43	–0.11	–1.77	0.076

Table S5. Motives, cooperation, and leadership evaluations by gender. Notes: Positive values of *d* and *t* indicate higher scores for women compared to men. *Leaders* state that they currently hold a professional leadership position or, if they are not working anymore, held one in the past. *Workers* report having work experience (but no leadership position). *Students* are either students or homemakers. *Leadership ratings (after social dilemma)* are difference values (ratings after dilemma minus baseline ratings). We measured *encouragement of cooperation* by counting all statements favoring cooperation (positive values) or selfishness (negative values). Count values are then log-transformed, aggregated using equal weights, and then aggregated over two independent observers ($r = 0.71$). Given that statements encouraging selfishness are more rare, we assume that they have a higher weight per statement in the conversation. By standardizing both types of statements separately before aggregating them, we assign an equal weight to both indices. We do not control for group size in any of the values reported in this table.

Dependent variable	Via	*n*	*β*	*z*	*P*	95% CI	
						Lower	Upper
Cooperation in social dilemmas							
Encouragement of cooperation	Functional affiliation motive	201	0.067	2.34	0.019	0.016	0.143
	Dysfunctional power motive	201	0.029	1.46	0.144	–0.003	0.082
Oil spills	Functional affiliation motive	201	–0.055	–2.08	0.037	–0.123	–0.015
	Dysfunctional power motive	201	–0.047	–2.13	0.034	–0.113	–0.013
Selfish business decisions	Functional affiliation motive	960	–0.033	–3.95	0.000	–0.053	–0.017
	Dysfunctional power motive	960	–0.066	–4.58	0.000	–0.096	–0.039
Leadership evaluation							
Leadership ratings (after social dilemma)	Functional affiliation motive	201	0.060	2.71	0.007	0.023	0.121
	Dysfunctional power motive	201	0.017	1.21	0.228	–0.006	0.055
Leadership ratings (in general)	Functional affiliation motive	486	0.002	0.32	0.750	–0.011	0.020
	Dysfunctional power motive	486	–0.009	–0.99	0.324	–0.033	0.008
Leadership position	Functional affiliation motive	961	–0.023	–1.33	0.185	–0.062	0.010
	Dysfunctional power motive	961	0.011	0.84	0.399	–0.014	0.041

Table S6. Indirect effects of gender via motives on cooperation and evaluations. Notes: Positive values of β and z indicate that women score higher on the dependent variable due to their average level on the mediating motive. We control for a dysfunctional affiliation motive and a functional power motive in all analyses. Additionally, we control for group size in the laboratory study. CI, bias-corrected confidence interval (10,000 bootstrap samples).

Original German item	English version
Instruction	
Bitte kreuzen Sie an, inwieweit die Aussagen im Arbeitskontext auf Sie zutreffen.	Please check to what extent these statements apply to you in the context of work.
Functional affiliation motive	
Ich wünsche mir, für meine verständnisvolle und kooperative Art gemocht zu werden.	I wish that people like me for being sympathetic and cooperative.
Bei Entscheidungen, die ich gegen den Willen anderer treffen muss, achte ich sehr darauf, nicht in eine Außenseiterposition zu geraten.	When I have to make decisions against the will of others, I pay close attention not to put myself in the position of an outsider.
Ich genieße es, mit anderen Menschen konstruktiv ein gemeinsames Ziel zu verfolgen.	I enjoy to constructively pursue a common goal with other people.
Gerade bei unpopulären Entscheidungen finde ich es besonders wichtig, viel Verständnis für diejenigen aufzubringen, die von solchen Entscheidungen betroffen sind.	Especially when making unpopular decisions, I find it particularly important to be appreciative of those who are affected by these decisions.
Dysfunctional affiliation motive	
Ich vermeide es um jeden Preis, Konflikte auszutragen, die das harmonische Miteinander in der Gruppe gefährden.	I avoid at all costs to engage in conflicts that jeopardize harmonious togetherness within the group.
Ich mache mir oft Sorgen, von anderen weniger gemocht zu werden, weil ich etwas Falsches sage. In solchen Momenten schweige ich lieber, als dass ich es riskiere, mit meiner Meinung anzuecken.	I often worry that others like me less for saying something wrong. In these moments I rather fall silent than risk to offend with my opinion.
Es ist mir wichtiger, Konflikte konstruktiv anzugehen, anstatt sie unter den Teppich zu kehren, nur um die Harmonie aufrecht zu erhalten. (reverse coded)	It is more important to me to approach conflicts constructively rather than sweeping them under the rug only to maintain harmony. (reverse coded)
Es ist mir sehr wichtig, von anderen akzeptiert zu werden. Deshalb sage ich manchmal Dinge, von deren Richtigkeit ich zwar nicht überzeugt bin, aber durch die ich gut dastehe.	It is very important to me to be accepted by others. Therefore I sometimes say things of which I am not convinced that they are right, but that make me look good.
Functional power motive	
Es stellt mich zufrieden, andere Menschen so in ihren Handlungen und Einstellungen zu beeinflussen, dass sie ungeahnte Fähigkeiten entdecken und herausfordernde Aufgaben bewältigen können.	It satisfies me to influence others in their actions and attitudes so that they discover unexpected capabilities and accomplish challenging tasks.
Es bereitet mir Freude, Verantwortung für eine übergeordnete Sache zu übernehmen, auch wenn das bedeuten kann, Rückschläge zu erfahren und Fehler eingestehen zu müssen.	It pleases me to take responsibility for a greater cause, even if that might involve experiencing setbacks and admitting mistakes.
Ich mag es, kontroverse Standpunkte zu vertreten, aber nur, solange es auf angemessene Art und Weise geschieht.	I like advancing controversial views, but only if it happens in an appropriate way.
Ich genieße es, durch die eigenen Einflussmöglichkeiten etwas beitragen zu können, das im Sinne übergeordneter Ziele steht.	I enjoy to contribute something through my channels of influence that is aligned with the greater good.
Dysfunctional power motive	
Ich genieße es, wenn andere meinen Rat oder meine Anweisungen einholen müssen, bevor sie handeln.	I enjoy it if others have to obtain my advice or instructions before they act.
Es gefällt mir, viel Macht und Einfluss zu haben, da es viele Menschen gibt, die man unter Kontrolle halten sollte.	It pleases me to have a lot of power and influence, because there are many people that you need to keep under control.
Es ist mir so wichtig, meine persönlichen Ziele zu erreichen, dass ich dafür auch andere Menschen benutzen würde.	It is so important for me to reach my personal goals that I would use other people for it.
Es ist ein schönes Gefühl, meinen gesellschaftlichen Status zu demonstrieren.	It is a nice feeling to demonstrate my social status.

Table S7. Wording of items measuring variants of affiliation and power motives. Notes: Items were translated to English and back translated to German. Discrepancies were resolved through discussion. Response scales range from 1 (*does not at apply at all*) to 6 (*fully applies*).

Scale		Heterotrait-monotrait ratio of inter-item correlations			
		1	2	3	4
1	Functional affiliation motive	—			
2	Dysfunctional affiliation motive	0.10	—		
3	Functional power motive	0.68	–0.47	—	
4	Dysfunctional power motive	–0.01	0.36	0.28	—

Table S8. Discriminant validity between affiliation and power motives. Notes: Hetereotrait-monotrait ratios of inter-item correlations compare average inter-item correlations within a scale to the average correlations of the items of that scale with the items of another scale ($n = 960$ to 961).

Item		Affiliation		Power	
		Functional	Dysfunctional	Functional	Dysfunctional
		λ	λ	λ	λ
Functional affiliation motive					
1	I wish that people like me for being sympathetic and cooperative.	**0.64**	0.19	0.38	0.29
2	When I have to make decisions against the will of others, I pay close attention not to put myself in the position of an outsider.	**0.36**	0.23	0.14	–0.04
3	I enjoy to constructively pursue a common goal with other people.	**0.39**	–0.14	0.15	–0.41
4	Especially when making unpopular decisions, I find it particularly important to be appreciative of those who are affected by these decisions.	**0.39**	–0.02	0.26	0.00
Dysfunctional affiliation motive					
1	I avoid at all costs to engage in conflicts that jeopardize harmonious togetherness within the group.	0.21	**0.54**	–0.20	0.03
2	I often worry that others like me less for saying something wrong. In these moments I rather fall silent than risk to offend with my opinion.	0.41	**0.70**	–0.31	0.10
3	It is more important to me to approach conflicts constructively rather than sweeping them under the rug only to maintain harmony. (reverse coded)	–0.17	**0.42**	–0.19	0.20
4	It is very important to me to be accepted by others. Therefore I sometimes say things of which I am not convinced that they are right, but that make me look good.	–0.06	**0.67**	–0.17	0.25
Functional power motive					
1	It satisfies me to influence others in their actions and attitudes so that they discover unexpected capabilities and accomplish challenging tasks.	0.13	–0.25	**0.42**	0.00
2	It pleases me to take responsibility for a greater cause, even if that might involve experiencing setbacks and admitting mistakes.	0.03	–0.20	**0.48**	0.07
3	I like advancing controversial views, but only if it happens in an appropriate way.	0.58	–0.42	**0.49**	0.12
4	I enjoy to contribute something through my channels of influence that is aligned with the greater good.	0.39	0.02	**0.66**	0.21
Dysfunctional power motive					
1	I enjoy it if others have to obtain my advice or instructions before they act.	0.02	0.12	0.17	**0.64**
2	It pleases me to have a lot of power and influence, because there are many people that you need to keep under control.	0.09	0.10	0.17	**0.80**
3	It is so important for me to reach my personal goals that I would use other people for it.	–0.11	0.06	0.06	**0.60**
4	It is a nice feeling to demonstrate my social status.	–0.21	0.32	0.12	**0.63**

Table S9. Four-dimensional measurement model for affiliation and power motives. Notes: Coefficients are standardized factor loadings from exploratory structural equation modeling. Hypothesized primary loadings on target motive variants are bolded. The hypothesized model fits the data well, RMSEA = 0.042, CFI = 0.98, $X^2/df = 2.68$, $X^2(62) = 166.1$, $P < 0.0001$ ($n = 961$).

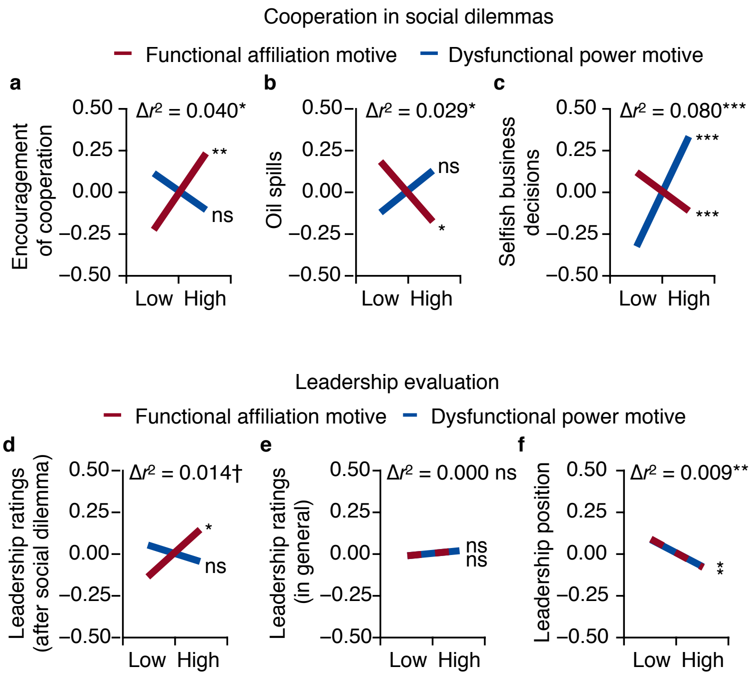

Figure S1. After accounting for an array of established predictors, the functional affiliation motive remains related to cooperation. In both studies, we control for personality (neuroticism, extraversion, openness, agreeableness, conscientiousness, fairness) and motivation to lead. (**a**, **b**, **d**) In the laboratory study (Settlers of Catan), we also control for implicit motives (need for affiliation, need for power, activity inhibition, as well as all three two-way interactions and the three-way interaction[58]), an achievement motive, and reasoning ability. (**a**, **b**) Motives relate to behaviour during a game of Settlers of Catan (n = 201). (**c**) Motives relate to selfish business decisions in a field survey (n = 960). (**d**) After the game of Settlers of Catan, all players rate each other on transformational leadership. (**e**) In the field survey, 739 peers rate the general leadership competence of 486 respondents. (**f**) Respondents state whether they hold a professional leadership position. See Supplementary Datasets 1 and 2 or https://osf.io/yt4qh/ for reliabilities, descriptive statistics, and intercorrelations of all variables used in each study. All values on y axes are z-standardized. Lines represent slopes from multiple regression analysis while also controlling for a dysfunctional affiliation motive and a functional power motive. Low/high ±1 s.d. *** $P < 0.001$, ** $P < 0.01$, † $P < 0.10$, two-sided t-tests. ns, not significant.

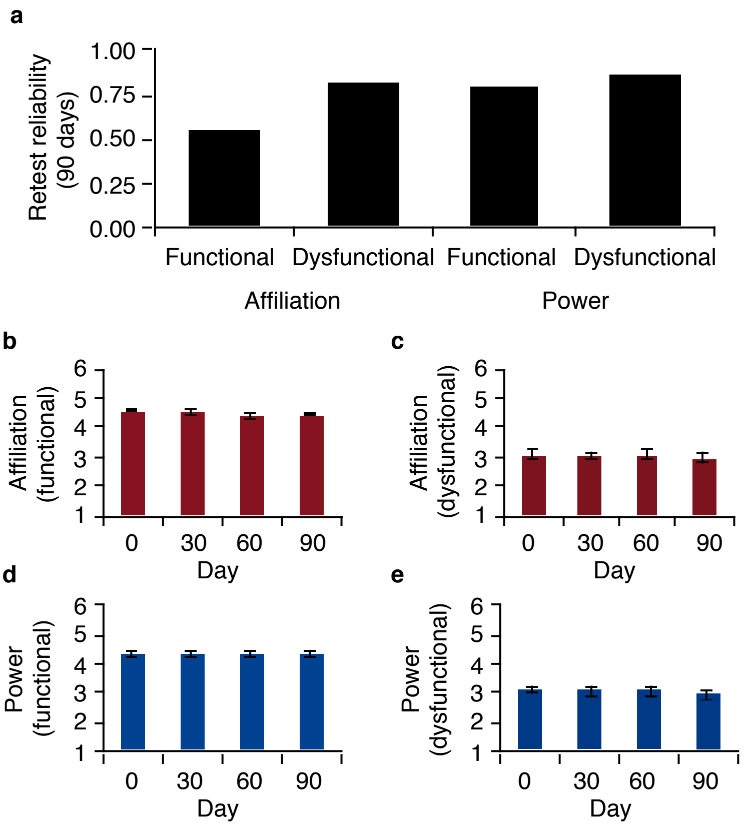

Figure S2. Stability of affiliation and power motives over a period of 90 days. (**a**) Intraclass correlations between the four measurement occasions every 30 days with measurement occasions nested within participants (n = 35 participants, 123 individual data points, 12% missing). (**b**-**e**) Mean values for each motive variant on each measurement occasion (n = 35 on day 0, n = 29 on day 30, n = 30 on day 60, n = 29 on day 90). Error bars indicate ± 1 s.e.m.

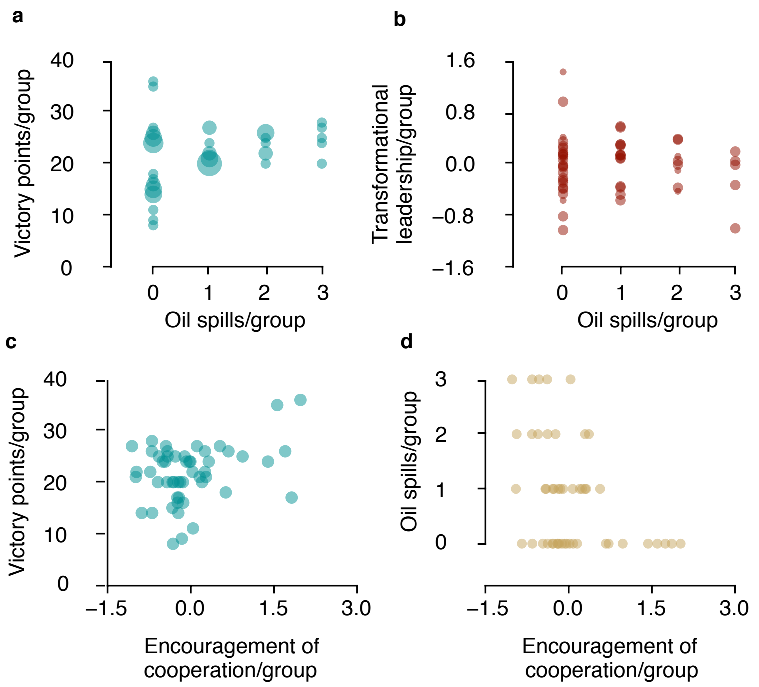

Figure S3. At the group level, high performance depends on encouragement of cooperation but not on oil spills (n = 52). (**a**) Group performance does not depend on oil spills (β = 0.09, P = 0.46). (**b**) Oil spills relate negatively to mutual ratings of transformational leadership (β = –0.21, P = 0.039). (**c**) Group performance depends on the average level of encouragement of cooperation in a group (β = 0.34, P = 0.0014). (**d**) In groups with high levels of encouragement of cooperation, fewer oil spills are caused (β = –0.41, P = 0.0014). This means that less fields are destroyed and, in turn, more resources will be available in the future. We use two-sided t-tests and control for group size in all analyses.

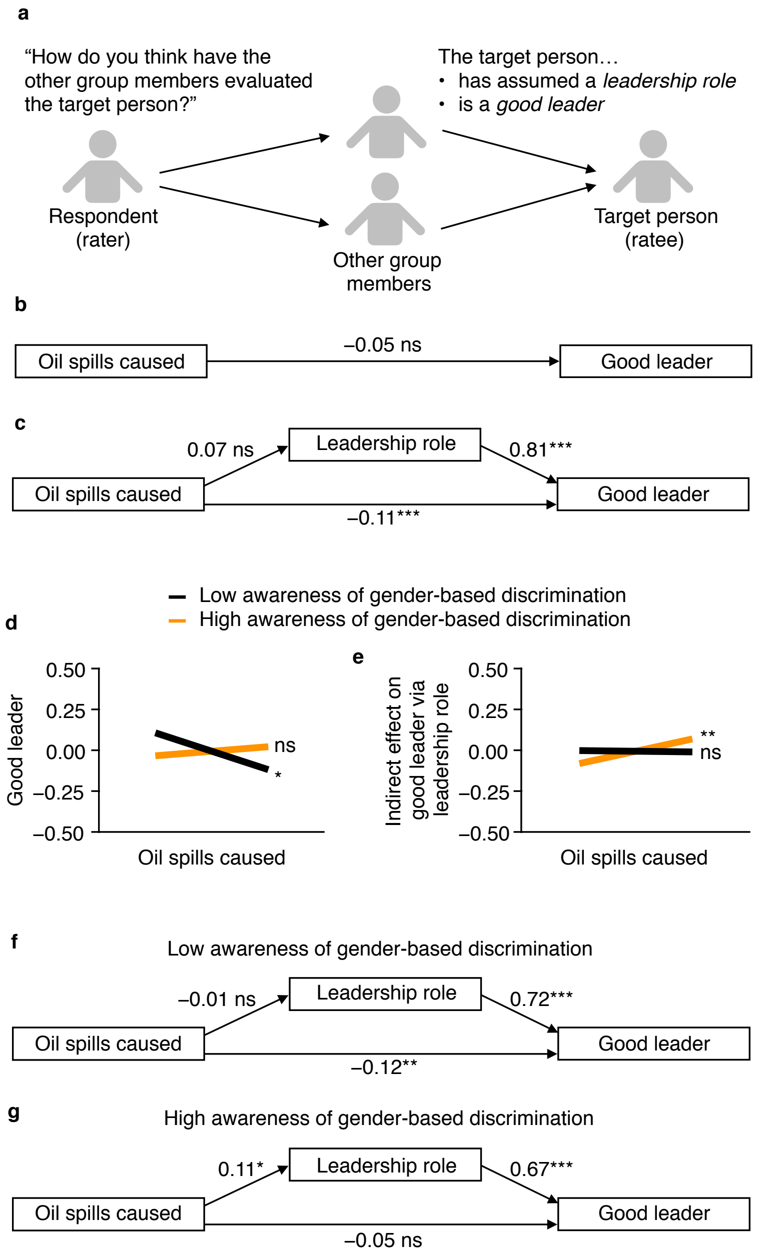

Figure S4. Respondents with high awareness of gender-based discrimination believe that others fail to disapprove of selfish behaviour. They believe that other group members think that (predominantly male) target persons who cause oil spills assume a leadership role and, in turn, are good leaders. (**a**) Schematic explaining the nature of second degree ratings (i.e., asking respondents to speculate about the thoughts of others[41]; Supplementary Information, Section 7). (**b**) Overall, no substantial relationship exists between the number of oil spills a target person causes and the guessed leadership rating that the target person receives from his/her group members after the game of Settlers of Catan. (**c**) Respondents hardly believe that group members evaluate those who cause oil spills as assuming a leadership role (n = 201 players). (**d**-**g**), However, respondents' awareness of gender-based discrimination itself does promote the belief that other group members show a stereotypical pattern of evaluation (n = 582 triads, displayed are conditional effects). (**d**, **f**) Respondents with *low* (−1 s.d.) awareness of gender-based discrimination believe that others disapprove of (predominantly male) players who cause oil spills. (**e**, **g**) In contrast, respondents with *high* (+1 s.d.) awareness of gender-based discrimination believe that other group members evaluate (predominantly male) players who cause oil spills as assuming a leadership role, and in turn as good leaders. All coefficients are z-standardized. *** $P < 0.001$, ** $P < 0.01$, * $P < 0.05$, two-sided t-tests. ns, not significant.

1. Construction and selection of questionnaire items to measure affiliation and power motives

1.1 Item development

As far as we know, no questionnaire exists that allows a separate assessment of functional and dysfunctional variants of affiliation and power motives. When conceptualizing the two variants of the affiliation motive, our most relevant source were McClelland and Burnham[1]. For the power motive, there is an array of theoretical arguments about its duality[2-5]. We considered ideas of McClelland and Burnham[1], Winter[3], and Magee and Langner[6] when we developed the initial item pool. We arrived at an initial item pool of 35 items (functional affiliation motive: 6 items, dysfunctional affiliation motive: 7 items, functional power motive: 9 items, dysfunctional power motive: 13 items). These numbers reflect the preponderance of the power motive in the literature as compared to the affiliation motive. We tolerated complex phrasing of an item if we deemed it useful for distinguishing the functional variant of a motive from its dysfunctional variant.

1.2 Sample for item selection

We then reduced the number of items from 35 to 16. For item selection, we used responses of the first n = 201 respondents of the field survey plus an additional n = 111 participants of the laboratory study (resulting in n = 312 individuals, 53% female, M_{age} = 26 y, s.d. = 14). After determining the final set of 16 items, only those items were presented to the remaining n = 758 respondents of the field survey.

1.3 Criteria for item selection

When we reduced the number of items from 35 to 16, we attempted to maximize the following criteria: (*i*) high correlation between an item and its scale, (*ii*) relatively lower correlation between an item and the other scales, particularly those with either the same motive (but a different variant) or the same variant (but a different motive), and (*iii*) meaningful coverage of the construct of interest. For example, criterion (*ii*) lead us to drop the item "if someone is well disposed to me I like to reward that with little somethings or favors" as an indicator for the dysfunctional affiliation motive given that it was substantially related with the dysfunctional power motive. As another example, criterion (*iii*) influenced our decision to drop the item "as a member of a group, I like representing it at public events" given that it did not unambiguously fit our definition of the functional variant of the affiliation motive. Supplementary Table S7 shows a list of the 16 items that we finally selected.

2. Psychometric evaluation of scales to measure affiliation and power motives

2.1 Unidimensionality of each motive variant

Unidimensionality is present if all questionnaire items that are selected as indicators for a particular construct covary only along one single dimension. This implies that no subgroups of indicators can be identified that covary along another dimension. One then infers that the observed indicators covary only because they measure the same underlying construct, i.e., the one that they are intended to measure. In this case, residual variance of each item is idiosyncratic[7]. Researchers interpret unidimensionality as an indication that they can aggregate single questionnaire items into a joint measurement model. When unidimensionality is present, they assume that this measurement model meaningfully represents the construct of interest[7]. In the framework of generalizability theory, this relates to the item facet of generalizability. Users of a measure want to be able to generalize from the sampled items to the unsampled item space representing the construct of interest.

We evaluated unidimensionality for each of the four motive variants. We applied minimum rank factor analysis[8] and calculated the ratio of the explained common variance (ECV) of the first factor compared to all remaining factors[9] using the program FACTOR[10]. We interpret ECV as the closeness of a scale to unidimensionality[11]. We used data from the field survey, because the sample was larger and more diverse as compared to the laboratory study. The ECV for the functional affiliation motive (78%) was slightly lower than the ECV for the dysfunctional affiliation motive (89%), the functional power motive (96%), and the dysfunctional power motive (90%). In all cases, parallel analysis[12] suggested a unidimensional solution.

2.2 Average inter-item correlations within each motive variant

The average correlation of all items of a scale with each other is an important property of a scale. For a construct that is conceptually narrow, high values are desirable whereas lower values are desirable for a broader construct[13]. There is a tradeoff known as bandwidth-fidelity dilemma[14] between redundancy (in the case of high correlations) versus low content saturation (in the case of low correlations) which is indicative of

a higher proportion of measurement error in the items[7]. For broad constructs such as motives, we do not expect inter-item correlations at the top end of the recommended 0.15 to 0.50 range[13]. When keeping the number of items constant, average inter-item correlations directly transform to Cronbach's alpha. In the field survey, we find low inter-item correlations for the functional affiliation motive (0.23, α = 0.53), intermediary values for the dysfunctional affiliation motive (0.34, α = 0.67) as well as the functional power motive (0.33, α = 0.66), and substantial inter-item correlations for the dysfunctional power motive (0.41, α = 0.74).

2.3 Discriminant validity between motive variants

We intended that each of the four motive variants measures a construct that is theoretically different from the other three motive variants. This needs to be reflected in relationships between the constructs that are lower than the relationships of the indicators within each construct. As a direct test of discriminant validity, we calculated the heterotrait-monotrait ratio of correlations[15]. This ratio compares the average correlations of items within a particular scale to the average correlations of the items of that scale with items of another scale. As presented in Supplementary Table S8, analyses of data from the field survey support discriminant validity of the four motive variants towards each other with all ratios being substantially lower than 1. The functional affiliation motive and the functional power motive have the highest overlap (0.68). We find lower ratios (|0.01| to |0.47|) for the remaining pairwise comparisons.

2.4 Measurement model

In order to test the hypothesized factorial structure when considering all four scales simultaneously, we perform exploratory structural equation modeling (ESEM). We choose ESEM over confirmatory factor analysis, because we expect many items to be related to more than one motive variant. In particular, we expect that some items not only capture variance of their focal motive variant, but also reflect variance of adjacent motive variants (i.e., the same motive or the same functionality). It is common that single items are imperfect indicators of a single construct[16, 17]. ESEM is suited to multidimensional questionnaires that contain imperfect indicators. ESEM integrates exploratory factor analysis and confirmatory factor analysis. Through factor rotation, ESEM allows researchers to target cross-loadings as close to zero as possible[18].

We use the robust weighted least square estimator implemented in M*plus* 7.3 to account for the ordinal response scale of the items. We collapse across extreme response categories so that each response category contains at least 5% of the responses to increase stability of estimation[19]. We evaluate model fit via root mean square error of approximation (RMSEA) and comparative fit index (CFI). Values lower than 0.06 for RMSEA and higher than 0.95 for CFI indicate good model fit.

Results show that a four-dimensional ESEM fits the data well, RMSEA = 0.042, CFI = 0.98 with a X^2/df of 2.68, $X^2(62) = 166.1$, $P < 0.0001$. As presented in Supplementary Table S9, primary factor loadings on the focal motive variant are all substantial, $\lambda_{\text{median}} = 0.57$, range = 0.36 to 0.80, $ts > 9.95$, $P < 0.0001$ whereas most cross-loadings on the adjacent motive variants are small, $\lambda_{\text{median}} = 0.17$, range = |0.00| to |0.58|.

2.5 Retest-reliability of each motive variant

We assessed retest-reliability of all scales to evaluate whether the scales capture stable interindividual differences in affiliation and power motives or whether they fluctuate heavily over time. If affiliation and power motives exhibit a considerable degree of stability, then it is more likely that they persistently influence individuals' choices and behaviours. We recruited $n = 35$ participants (80% female, $M_{\text{age}} = 26$ y, s.d. = 7, 91% students) who completed the scales for affiliation and power motives four times within a period of 3 months (30-32 days between each measurement occasion) resulting in a total number of 123 (of 140) data points (12% missing). We conducted multilevel analysis with the four measurement occasions (level 1) nested within participants (level 2). In this analysis, the intraclass correlation coefficient (ICC) of the null model reflects the amount of variance that can be attributed to individual participants in relation to the total amount of variance observed over the period of 3 months (higher ICCs indicate higher stability over time). Stabilities are relatively high with ICCs ranging between 0.55 and 0.86 (functional affiliation motive: 0.55, dysfunctional affiliation motive: 0.81, functional power motive: 0.79, dysfunctional power motive: 0.86, Supplementary Fig. S2).

3. Modifications to the procedure of the game of Settlers of Catan

3.1 Standardization

We wanted to be able to compare individuals' behaviour during the game between all groups. For this purpose, we took multiple steps to standardize the procedure of the game. First, instead of actual dice, we used chips with printed numbers on them. Instead of rolling the dice, the player whose turn it was turned the chip. The numbers on the chips substituted the numbers on a dice. We determined the order of the numbers on the chips at random and then modified the numbers itself so that each number (from 2 to 12, representing two dices with

6 numbers each) occurred in the frequency that would be expected on average. Given that participants did not know how long the game lasted, they were unable to exploit this fact. We divided the chips into multiple piles and provided extra chips (exceeding the actual duration of the game) so that participants could not predict the duration of the game from the height of the pile of chips. We prepared another set of chips to determine type and location of an oil spill. We allowed a maximum of 3 oil spills (Supplementary Fig. S3). Moreover, we simplified the set of development cards so that only knight cards and monopoly cards were available. Both occurred in identical proportions (so that each type had a probability of 50%). Developmental cards were provided in the same order in each group.

3.2 Information sheet with hints

We provided all participants with a document containing the most important information, including advice about useful strategies for playing the game. This step was designed to advance all participants to a similar level with regard to their understanding of basic principles of the game.

3.3 Goal of the game

The original rules of the game define that the game always has exactly one winner (i.e., the person who first accumulates 12 victory points). We eliminated this rule. Instead, we informed participants that the game ended after a fixed amount of rounds which we kept secret. Furthermore, we stated that the goal of the game is to populate the island as well as possible. We pointed out that participants could freely choose if they would like to support the other members of their group or even obstruct them. We suggested that there was no one "right" strategy. We informed participants that they received a payoff that was either based on their individual victory points or on the group average (decided by a coin flip after the game). On the information sheet, we advised participants that it might be profitable to collaborate with the other group members.

4. Utility of cooperation in the game of Settlers of Catan

The interpretation of the main results of this paper—i.e., how motives shape cooperation and how motives affect leadership evaluations—somewhat depends on the utility of cooperation under the specific circumstances of our studies. Is it better to cooperate or to act selfishly in the situations that we examine in our studies? In this section, we discuss this question with respect to the laboratory study. In the field survey, we believe that most people agree that good leaders avoid the kinds of selfish business decisions measured there (see ref. 20 for the wording of the scenarios that we used).

4.1 Utility of cooperation for the individual

As a proxy for the utility of cooperation in the laboratory study, we use the number of victory points which participants earned in the game of Settlers of Catan. Victory points, in turn, determine the financial payoff to participants. Given that an actual coin toss after the game decided whether we payed participants based on their own victory points or on the average number of victory points of all group members, we calculated an expected value for the individual payoff that averages both types of payoff.

We find that causing an oil spill is actually profitable for the individual who causes the oil spill, $\beta = 0.21$, $t = 3.12$, $P = 0.0021$. Oil spills are a direct consequence of the use of oil, which buys resources and buildings so that victory points are earned faster than without using oil. Translated to raw values, this means that each oil spill earns the individual who causes it an average of €0.40 (s.e.m. = 0.13). Such individual benefits associated with oil spills are to be expected, given that the underlying oil use offers powerful short-term advantages in the game that are meant to be similar to the real-world phenomena that are modeled in the game[21]. It is characteristic for one-shot public goods games in general that acting selfishly results in a higher payoff for the selfish individual.

4.2 Utility of cooperation for the group

In contrast to benefits for the individual, causing oil spills does not increase the sum of the victory points of all members of a group, $\beta = 0.09$, $t = 0.74$, $P = 0.46$ ($n = 52$ groups, Supplementary Fig. S3). On a descriptive level, the data show that the two highest performing groups are able to achieve such performances without causing a single oil spill, indicating there are ways to success beyond oil. When analyzing individual data ($n = 201$), we find a non-significant negative relationship between oil spills and the average number of victory points of the other group members, $\beta = -0.08$, $t = 1.15$, $P = 0.25$.

Furthermore, victory points do not reflect the future loss of resources that is due to the permanent damage from oil spills as defined by the rules of the game. As participants did not know how long the game lasts, they could not objectively predict how severely this future loss of resources would affect them and their group members. Anecdotal evidence from observing the groups suggests that group members never reacted positively

whenever one of the players decided to cause an oil spill. It seems as if the other group members understood that they had to pay the price for the behaviour of the selfish individual. In fact, oil spills relate negatively to the group mean of ratings of transformational leadership, β = –0.21, t = 2.13, P = 0.039 (n = 52, Supplementary Fig. S3).

In contrast to oil spills, verbal encouragement of cooperation was an unambiguously positive behaviour. Supplementary Fig. S3 shows that the group mean of verbal encouragement of cooperation positively relates to group performance both in terms of victory points, β = 0.34, t = 3.39, P = 0.0014, as well as in terms of future availability of resources, β = 0.41, t = 3.38, P = 0.0014 (or, in other words, in terms of avoidance of oil spills).

In summary, these findings suggest that oil spills yield small benefits for an individual in terms of financial profit. For the whole group, however, oil spills do not increase financial profit (based on aggregate victory points) but decrease future availability of resources (which unfolds more and more, the longer the games lasts). Two groups were able to achieve outstanding performance without causing a single oil spill. Verbal encouragement of cooperation is effective both with regard to financial profit and future availability of resources.

5. Awareness of gender-based discrimination moderates evaluation of oil-spill causing players

5.1 Analytical approach

This section provides more details for the analysis presented in Fig. 4c-f, i.e., whether awareness of gender-based discrimination links to systematic differences in the tendency to rate oil-spill causing players as assuming a leadership role and, in turn, as transformational leaders. We use the process macro for SPSS (v. 2.16.1) for mediation and moderation analyses. In all multiple regression analyses, we include the same control variables as throughout the manuscript (group size: 3 or 4 players, baseline values of all ratings). In all analyses of moderated mediation, we specify the least restricted models which include interaction terms for all three paths that are displayed in Fig. 4b, e, f.

5.2 Interaction effects

In addition to the findings already reported in the main text, here we only report interaction terms. In an unmediated moderation model predicting ratings of *transformational leadership* by the number of oil spills a ratee causes, the interaction with a respondent's awareness of gender-based discrimination is β = 0.06, t = 1.98, P = 0.048 (Fig. 4c). In a mediated moderation model predicting ratings of *assumed leadership role* by the number of oil spills a ratee causes, the interaction with rater's awareness of gender-based discrimination is β = 0.10, t = 2.95, P = 0.003. When predicting ratings of *transformational leadership* by *assumed leadership role*, the interaction with a respondent's awareness of gender-based discrimination is β = –0.10, t = –3.24, P = 0.0013. When predicting ratings of *transformational leadership* by the number of oil spills a ratee causes, the interaction with a respondent's awareness of gender-based discrimination is β = 0.05, t = 1.75, P = 0.081. The resulting differences in conditional regression weights are displayed in Fig. 4e, f.

6. Data analysis using multilevel analysis

The data from the laboratory study possess a multilevel structure in that (*i*) individual participants are nested within groups and (*ii*) ratings are nested within ratees. Multilevel analysis might therefore appear to be the method of choice for data analysis. In this section, we first provide reasons why we do not choose multilevel analysis in our particular cases. Second, we still report results using multilevel analysis for the interested reader. Our findings converge with those using standard regression analysis, even though some coefficients are slightly smaller.

6.1 Modeling participants as being nested within groups using multilevel analysis

6.1.1 Assumed necessity of multilevel analysis. In a typical scenario for multilevel analysis, being member in a particular group (e.g., a class at school) acts as a third variable that—over an extended period of time—potentially influences multiple variables of all members of that group (e.g., a particular teacher influences motivation and performance of all students of one class). Furthermore, group membership is often not randomly distributed (e.g., students of one particular school often share similar socio-demographic backgrounds). Group membership can thereby cause or represent spurious correlations between variables on the level of the individual members of a group which would otherwise be unrelated. Due to this kind of impact of group membership, it is often appropriate to account for group membership using multilevel analysis.

In our case, however, we do not expect this impact of group membership to be present. Group membership was temporarily determined so that it was only effective during the time of interaction in the laboratory. Group members did not know each other before meeting in the laboratory (the average degree of familiarity between group members was M = 1.2, s.d. = 0.6, on a scale of 1 to 6). Furthermore, our software composed groups basically at random by offering available time slots to participants via automated emails. In these circumstances, we interpret all similarities within groups as being a consequence of the effect of group members' characteristics on group interaction (group members' characteristics determine who influences others and who accepts being influenced). We do not interpret similarities within groups as being a consequence of third variables at the group level. Whereas multilevel analysis partials out similarities within groups from analyses on the level of individuals, we believe that in this particular case, similarities within groups constitute valid portions of variance that are to be explained by independently measured characteristics of participants (such as their motives). In our case, participants' characteristics (and their effect on group interaction) are the predominant factor determining behaviour (considering that there are no discernible third variables at the group level). We prefer to interpret similarities within groups as being the consequence of group members' characteristics rather than being the consequence of arbitrary third variables associated with group membership. From this perspective, standard regression analysis serves our purpose better than multilevel analysis. For the interested reader, we still report the results using multilevel analysis. Our findings converge with those using standard regression analysis.

6.1.2 Null models for all dependent variables from the laboratory study using multilevel analysis. First, we report null models for all three dependent variables of the laboratory study. For each dependent variable, null models allow to determine whether the variability between groups constitutes a significant portion of the total variability. This indicates that members of a group are more similar to each other compared to members of other groups. The intraclass correlation coefficient (ICC) is a measure of similarity within groups. As throughout the manuscript, we include group size (3 vs. 4) as a control variable. For encouragement of cooperation, we find an ICC of 0.31 based on a significant amount of variance between groups, σ^2 = 0.31, Wald's Z = 3.16, P = 0.0016. For oil spills, we find an ICC of 0.00 indicating that we cannot systematically attribute portions of variance in oil spills to group membership. For ratings of transformational leadership after the game (controlling for baseline values), we find an ICC of 0.09 based on a not significant amount of variance between groups, σ^2 = 0.04, Wald's Z = 1.28, P = 0.20.

6.1.3 Relationship between motives and encouragement of cooperation using multilevel analysis. Second, based on these ICCs, we include affiliation and power motives as predictors on level 1 with encouragement of cooperation as the dependent variable. Similar to the findings displayed in Fig. 1a, the functional affiliation motive positively relates to encouragement of cooperation, β = 0.23, t = 3.64, P = 0.0004, whereas the dysfunctional power motive negatively relates to encouragement of cooperation, β = –0.17, t = –2.87, P = 0.005. This finding indicates that multilevel analysis yields an identical pattern of results in this case as compared to standard multiple regression analysis.

6.2 Modeling ratings as being nested within ratees using multilevel analysis

6.2.1 Assumed necessity of multilevel analysis. For the analyses reported in Fig. 4c-f, Supplementary Fig. S4, and Supplementary Information, Section 5, one might assume that this is a case to apply multilevel analysis because raters are nested within ratees (each ratee receives ratings from the other 2-3 individuals who are in the same group as the ratee). In contrast to standard regression analysis, multilevel analysis accounts for dependencies between units of analysis that are due to membership in a higher level entity (e.g., multiple ratings belonging to a single ratee). By accounting for dependencies in individual observations, multilevel analysis avoids underestimation of standard errors. It achieves this by estimating a separate model for each higher level entity (here: for each ratee).

However, we argue that in the present case, this complexity is not necessary and might even reduce statistical power. In our opinion, multilevel analysis is not necessary in this case because all observations used to estimate our parameter of interest—the prediction of leadership ratings by the interaction between a respondent's awareness of gender-based discrimination and the number of oil spills a target person causes—are actually independent from each other once the main effect of oil spills is included in the regression model. This is because awareness of gender-based discrimination was measured *before* raters were assigned to ratees. Ratees therefore cannot have influenced raters' awareness of gender-based discrimination.

6.2.2 Assumed impact of multilevel analysis on statistical power. In the present case of analysis, we furthermore argue that multilevel analysis reduces statistical power because it controls for the mean level on the dependent variable of each ratee. From this follows that the total amount of error variance is increased by introducing error variance from group composition (via group composition, raters are assigned to ratees) which is a type of sampling error. For instance, imagine a group with a ratee causing multiple oil spills and three group members who—by chance—all have high awareness of gender-based discrimination and who all (as a consequence of

their awareness of gender-based discrimination) rate that particular target person as being influential. In comparison to the other ratees, multilevel analysis would model the extremity of these ratings as being a mere effect of the ratee for whom multilevel analysis estimates a unit-specific error term (whereas standard regression analysis treats these ratings as valid portions of variance). At this point, multilevel analysis models valid portions of variance (which could be used for comparing respondents rating *this* ratee to respondents rating *another* ratee) as error variance. From this follows that a unit contributes towards decreasing statistical power if both of the following conditions are met: (*i*) the distribution of the variable of interest (here: awareness of gender-based discrimination) deviates from the whole sample (due to sampling error that occurred during group composition where raters are assigned to ratees) and (*ii*) the unit mean on the dependent variable (of one particular ratee) deviates from the grand mean on the dependent variable (over all ratees) so that a unit-specific error term is modeled. Multilevel analysis does not compare each rater to all other raters who participated in the study, but only to those raters who rate the same ratee. Compared to the whole sample, raters in the same group can be particularly similar or dissimilar to one another due to random error that occurred during group composition. Error variance from random composition of groups is thereby introduced when applying multilevel analysis (which accounts for the hierarchical structure of the data) as compared to standard regression analysis (which ignores the hierarchical structure of the data). Increased error variance, in turn, reduces statistical power so that we expect statistical power to be reduced when using multilevel analysis. Even though we have argued that we believe multilevel analysis to be neither necessary nor beneficial (in terms of statistical power) in our particular case, we still report the results using multilevel analysis for the interested reader. Our findings converge with those using standard regression analysis, even though some coefficients are slightly smaller.

6.2.3 Analytical approach and null models. First, we report null models for both types of ratings displayed in Fig. 4, i.e., the dependent variable *transformational leadership* as well as the mediator variable *assumed leadership role*. As throughout the membership, we control for group size (3 vs. 4) and baseline ratings. For *transformational leadership*, we find an ICC of 0.13 based on a significant amount of variance between ratees, $\sigma^2 = 0.08$, Wald's $Z = 2.69$, $P = 0.007$. For *assumed leadership role*, we find an even higher ICC of 0.35 based on a substantial amount of variance between ratees, $\sigma^2 = 0.26$, Wald's $Z = 5.87$, $P < 0.0001$.

6.2.4 Examining the moderating role of awareness of gender-based discrimination on the evaluation of oil-spill causing players using multilevel analysis. Second, based on these ICCs, we include (*i*) respondent's awareness of gender-based discrimination, (*ii*) the number of oil spills a ratee causes, and (*iii*) the interaction between both as predictors on level 1 with *transformational leadership* as the dependent variable. Very similar to the finding from standard regression analysis displayed in Fig. 4c and reported in Supplementary Information, Section 5, the interaction term explains a significant share in transformational leadership, $\beta = 0.06$, $t = 1.97$, $P = 0.049$, indicating that respondents with higher awareness of gender-based discrimination evaluate oil-spill causing group members more positively as compared to respondents with lower awareness of gender-based discrimination. We repeat this analysis with *assumed leadership role* as the dependent variable. The interaction term again explains a significant share in *assumed leadership role*, $\beta = 0.08$, $t = 2.30$, $P = 0.022$ which, however, appears to be slightly lower than the finding from standard regression analysis displayed in the first paths of Fig. 4e, f and reported in Supplementary Information, Section 5. These findings indicate that multilevel analysis yields a similar pattern of results with respect to the moderating effects of awareness of gender-based discrimination as compared to standard regression analysis.

7. Potential explanations for the moderating role of awareness of gender-based discrimination on evaluation of oil-spill causing players

7.1 Evaluation of selfish behaviour in Western cultures

In a completely cooperative world, people would clearly disapprove of selfish behaviour (such as causing an oil spill). However, under the norm of self-interest which prevails in Western cultures[22], there is no unanimous disapproval of selfish behaviour in leaders. In contrast, selfish behaviour can even be interpreted as a signal for status[23, 24]. Most people tend to believe that leaders are masculine[25], dominant[26], and sometimes tyrannical[27]. People-oriented leaders are perceived as less effective in task performance[28], even though this is not necessarily the case[29]. Trustworthy leaders are perceived as less dominant[30]. Dominant individuals are perceived as competent[31]. Competent individuals tend to be perceived as less warm[32]. Some people even endorse selfish leaders[33].

7.2 How awareness about stereotypes may inadvertently reproduce stereotypes

We identify three explanations why awareness of gender-based discrimination affects leadership ratings. First, people who are aware of gender-based discrimination know at least some stereotypes about leadership because

these stereotypes are very pervasive[25]. Leadership stereotypes can be seen as societal standards. Being aware of a societal standard often correlates with internalizing it as a personal standard[34], which can lead to behavioural change[35, 36]. Second, awareness of gender-based discrimination reflects some kind of *extrapersonal knowledge* which has been shown to influence automatic mental associations[37], which in turn predict behaviour, particularly in socially sensitive domains[38]. Third, awareness of gender-based discrimination might elicit ratings that are conform with leadership prototypes, even if such prototypes contradict the personal opinion of the rater[39, 40].

7.3 How awareness may shape what we think what others think

7.3.1 Theoretical background. It is important to understand why awareness of gender-based discrimination might promote stereotypical patterns of evaluation. Such an understanding might be used to develop remedies that change stereotypical patterns of evaluation. Therefore, we test the explanations outlined above by asking raters about their beliefs about ratings from others. If the explanations above apply, then individuals with high awareness of gender-based discrimination should believe that other people evaluate selfish behaviour in the same way as they do, i.e., that other people interpret oil spills as a form of leadership behaviour by which a target person assumes a leadership role, which in turn raises other people's leadership evaluations of that target person (see Supplementary Fig. S4 for a schematic).

7.3.2 Method. We measured *respondents' beliefs about their group members' ratings* by asking respondents to speculate about the ratings that the other two group members provided about the fourth group member (Supplementary Fig. S4). This type of question is based on Keynes' guessing game (also known as the *beauty contest* game[41]). We asked respondents to "please estimate how the other group members presumably evaluated [first name of the fourth group member]. Try to take the perspective of the other group members." Supplementary Fig. S4 displays the two items that we used. We motivated respondents to respond accurately by offering a small incentive based on the actual accuracy of their guess (up to €0.20 per rating), which was calculated and paid at the end of the study.

7.3.3 Assumptions about others' evaluation of oil-spill causing players. In the first step, we analyze whether a respondent—irrespective of his/her awareness of gender-based discrimination—believes that a target person who causes oil spills is seen as a good leader by the other two group members and if this relationship is mediated by respondent's belief that group members think that the oil-spill causing target person assumes a leadership role (n = 201, aggregated within target persons).

The standardized total effect of a target person's oil spills on guessed *good leader* ratings is not significant, β = –0.05, t = –0.95, P = 0.34 (Supplementary Fig. S4). The path from target person's oil spills to guessed *assumed leadership role* is not significant either, β = 0.07, t = 1.23, P = 0.22. The path from guessed *assumed leadership role* to guessed *good leader* ratings is strong, β = 0.81, t = 21.08, P < 0.0001. The indirect path from target person's oil spills to guessed *good leader* ratings via *assumed leadership role* is not significant, β = 0.059, z = 1.22, P = 0.22, 95% CI [–0.026, 0.160]. The direct path from target person's oil spills to guessed *good leader* ratings is negative and significant, β = –0.11, t = –3.55, P = 0.0005 (Supplementary Fig. S4). These analyses indicate that respondents (irrespective of their awareness of gender-based discrimination) do not generally believe that an oil-spill causing player receives positive ratings from the other group members.

7.3.4 Moderating role of awareness of gender-based discrimination on assumptions about others' evaluation of oil-spill causing players. In the second step, we analyze whether respondent's awareness of gender-based discrimination moderates respondent's beliefs about the other two group members' opinion of the target person (Supplementary Fig. S4) in a similar way as it does for ratings from respondents themselves (Supplementary Information, Section 5). We use the disaggregated data set for these analyses with each individual triad being a data point, resulting in n = 582 triads (by *triad* we refer to data points that reflect a respondent's belief about his/her group members' opinion of a target person, Supplementary Fig. S4). We find that respondent's awareness of gender-based discrimination interacts with the number of oil spills a target person causes in predicting respondent's belief whether the other group members rate the target person as being a good leader, β = 0.07, t = 2.14, P = 0.033. The conditional effect of a target person's oil spills on guessed *good leader* ratings is negative for respondents with low (–1 s.d.) awareness of gender-based discrimination, β = –0.11, t = –2.16, P = 0.032, whereas it is not significant for respondents with high (+1 s.d.) awareness of gender-based discrimination, β = 0.03, t = 0.62, P = 0.53 (Supplementary Fig. S4). In this analysis, the main effects of oil spills, β = –0.04, t = –1.19, P = 0.24, and awareness of gender-based discrimination, β = –0.00, t = –0.04, P = 0.97, are not significant. These findings suggest that only respondents with *low* awareness of gender-based discrimination believe that their group members disapprove of a target person who causes oil spills. Respondents with *high* awareness of gender-based discrimination, on the other hand, believe that their group members are neutral towards a (most likely male) target person who causes oil spills.

We also analyze whether the moderating effect of respondent's awareness of gender-based discrimination is reflected in a positive indirect effect via respondent's belief that group members think that a target person

who causes oil spills assumes a leadership role (moderated mediation, Supplementary Fig. S4). We use the least restricted model in which includes interaction terms for all three paths (reflected in different conditional regression coefficients for all paths, see bottom panels of Supplementary Fig. S4). The effect of the interaction between respondent's awareness of gender-based discrimination and target person's oil spills on guessed *assumed leadership role* ratings is marginally significant, β = 0.06, t = 1.80, P = 0.072. The effect of the interaction between respondent's awareness of gender-based discrimination and guessed *assumed leadership role* ratings on guessed *good leader* ratings is not significant, β = –0.03, t = –1.26, P = 0.21. The effect of the interaction between respondent's awareness of gender-based discrimination and target person's oil spills on guessed *good leader* ratings is not significant either, β = 0.03, t = 1.27, P = 0.17. The conditional indirect effect of target person's oil spills on guessed *good leader* ratings via guessed *assumed leadership role* ratings is not significant for respondents with low (–1 s.d.) awareness of gender-based discrimination, β = –0.004, s.e.m. = 0.037, 95% CI [–0.077, 0.072] whereas it is significant for respondents with high (+1 s.d.) awareness of gender-based discrimination, β = 0.075, s.e.m. = 0.028, 95% CI [0.025, 0.134] (Supplementary Fig. S4).

More specifically, respondents with low (–1 s.d.) awareness of gender-based discrimination do not believe that their group members think that those who cause oil spills assume a leadership role, β = –0.01, t = –0.11, P = 0.92. Instead, they believe that their group members think that those who cause oil spills are bad leaders, β = –0.12, t = –3.14, P = 0.0018 (Supplementary Fig. S4). Respondents with high (+1 s.d.) awareness of gender-based discrimination, in contrast, believe that their group members think that those who cause oil spills *do* assume as leadership role, β = 0.11, t = 2.53, P = 0.012, and do not believe (to a statistically significant extent) that their group members think that those who cause oil spills are bad leaders, β = –0.05, t = –1.63, P = 0.104 (Supplementary Fig. S4). Altogether, these findings suggest that awareness of gender-based discrimination is accompanied by the belief that others make evaluations somewhat in favor of those (predominantly male) individuals who cause oil spills. In short, awareness of gender-based discrimination links to the belief that others make stereotypical evaluations.

7.3.5 Moderating role of awareness of gender-based discrimination on assumptions about others' evaluation of oil-spill causing players using multilevel analysis. Even though we have argued in Supplementary Information, Section 6 that we believe that multilevel analysis is neither necessary nor beneficial (in terms of statistical power) in our particular case, we still report the results using multilevel analysis for the interested reader. The interaction effects that were almost not significant using standard regression analysis are not significant using multilevel analysis. As we argue above, this difference between multilevel analysis and standard regression analysis is particularly present for dependent variables with high ICCs.

First, we report null models for both types of ratings displayed in Supplementary Fig. S5, i.e., the dependent variable guessed *good leader* ratings as well as the mediator variable guessed *assumed leadership role*. For guessed *good leader* ratings, we find an ICC of 0.25 based on a substantial amount of variance between ratees, σ^2 = 0.18, Wald's Z = 4.68, P < 0.0001. For guessed *assumed leadership role*, we find an ICC of 0.36 also based on a substantial amount of variance between ratees, σ^2 = 0.26, Wald's Z = 5.96, P < 0.0001.

Second, based on these ICCs, we include (*i*) respondent's awareness of gender-based discrimination, (*ii*) the number of oil spills a ratee causes, and (*iii*) the interaction between both as predictors on level 1 with guessed *good leader* ratings as the dependent variable. Similar to the finding from standard regression analysis displayed in Supplementary Fig. S4 and reported above, the interaction term explains a marginally significant share in guessed *good leader* ratings, β = 0.06, t = 1.88, P = 0.061, indicating that respondents with high awareness of gender-based discrimination believe that their group members think that oil-spill causing group members are good leaders. We repeat this analysis with guessed a*ssumed leadership role* as the dependent variable. The interaction term explains a share in guessed *assumed leadership role* that is not statistically significant, β = 0.04, t = 1.19, P = 0.24, and which is somewhat lower than the finding from standard regression analysis displayed in the first paths of the bottom two panels of Supplementary Fig. S4.

These findings indicate that multilevel analysis yields a relatively similar pattern of results as compared to standard regression analysis with respect to the moderating effect of awareness of gender-based discrimination on the relationship between a target person's oil spills and respondent's belief about the ratings from the other group members. However, the interaction effects on the mediating mechanism that were almost not significant using standard regression analysis are not significant using multilevel analysis. As we argue above, this difference between multilevel analysis and standard regression analysis is particularly present for dependent variables with high ICCs.

In summary, the results using standard regression analysis are in line with our explanations why awareness of gender-based discrimination affects leadership evaluations. Individuals with high awareness of gender-based discrimination believe that other people interpret selfish behaviour as a form of leadership behaviour, enabling one to assume a leadership role, which in turn is characteristic of good leaders. In short, awareness of gender-based discrimination links to the belief that others do not disapprove of selfish behaviour, such as causing oil spills.

7.4 Conclusion

These results highlight potential risks from increasing awareness of gender-based discrimination. Instead of reducing discrimination, the opposite can happen. Therefore, it seems helpful if out-dated stereotypes about leadership will not be reproduced more than necessary, but rather be substituted by new messages that contradict old-fashioned views of leadership. We recommend that organizations appreciate cooperativeness and pay close attention to motives that shape cooperation in and beyond social dilemmas.

References

1. McClelland, D. C. & Burnham, D. H. Power is the great motivator. *Harvard Bus. Rev.* **25**, 159-166 (1976).
2. Wang, F. & Sun, X. Absolute power leads to absolute corruption? Impact of power on corruption depending on the concepts of power one holds. *Eur. J. Soc. Psychol.* **46**, 77-89 (2016).
3. Winter, D. G. Taming power: generative historical consciousness. *Am. Psychol.* **71**, 160-174 (2016).
4. Sturm, R. E. & Antonakis, J. Interpersonal power: a review, critique, and research agenda. *J. Manag.* **41**, 136-163 (2015).
5. McClelland, D. C. The two faces of power. *J. Int. Aff.* **24**, 29-47 (1970).
6. Magee, J. C. & Langner, C. A. How personalized and socialized power motivation facilitate antisocial and prosocial decision-making. *J. Res. Pers.* **42**, 1547-1559 (2008).
7. John, O. P. & Benet-Martínez, V. in *Handbook of Research Methods in Social and Personality Psychology* (eds Reis, H. T. & Judd, C. M.) 339-369 (Cambridge Univ. Press, New York, 2000).
8. Ten Berge, J. M. F. & Kiers, H. A. L. A numerical approach to the approximate and the exact minimum rank of a covariance matrix. *Psychometrika* **56**, 309-315 (1991).
9. Ten Berge, J. M. F. & Sočan, G. The greatest lower bound to the reliability of a test and the hypothesis of unidimensionality. *Psychometrika* **69**, 613-625 (2004).
10. Lorenzo-Seva, U. & Ferrando, P. J. FACTOR 9.2: A comprehensive program for fitting exploratory and semiconfirmatory factor analysis and IRT models. *Appl. Psych. Meas.* **37**, 497-498 (2013).
11. Sijtsma, K. On the use, the misuse, and the very limited usefulness of Cronbach's alpha. *Psychometrika* **74**, 107-120 (2009).
12. Timmerman, M. E. & Lorenzo-Seva, U. Dimensionality assessment of ordered polytomous items with parallel analysis. *Psychol. Methods* **16**, 209-220 (2011).
13. Clark, L. A. & Watson, D. Constructing validity: basic issues in objective scale development. *Psychol. Assessment* **7**, 309-319 (1995).
14. Cronbach, L. J. & Gleser, G. C. *Psychological Tests and Personnel Decisions* (Univ. of Illinois Press, Oxford, 1965).
15. Henseler, J., Ringle, C. M. & Sarstedt, M. A new criterion for assessing discriminant validity in variance-based structural equation modeling. *J. Acad. Market. Sci.* **43**, 115-135 (2015).
16. Marsh, H. W. *et al.* A new look at the Big Five factor structure through exploratory structural equation modeling. *Psychol. Assessment* **22**, 471-491 (2010).
17. Morin, A. J. S., Arens, A. K. & Marsh, H. W. A bifactor exploratory structural equation modeling framework for the identification of distinct sources of construct-relevant psychometric multidimensionality. *Struct. Equ. Modeling* **23**, 116-139 (2016).
18. Morin, A. J. S., Arens, A. K., Tran, A. & Caci, H. Exploring sources of construct-relevant multidimensionality in psychiatric measurement: a tutorial and illustration using the composite scale of morningness. *Int. J. Method Psych.* **25**, 277-288 (2016).
19. Brown, M. B. & Benedetti, J. K. On the mean and variance of the tetrachoric correlation coefficient. *Psychometrika* **42**, 347-355 (1977).
20. Ashton, M. C. & Lee, K. The prediction of Honesty-Humility-related criteria by the HEXACO and Five-Factor Models of personality. *J. Res. Pers.* **42**, 1216-1228 (2008).
21. Griswold, A. High-stakes game of oil use. *Proc. Natl Acad. Sci. USA* **110**, 2685 (2013).
22. Miller, D. T. The norm of self-interest. *Am. Psychol.* **54**, 1053-1060 (1999).
23. Anderson, C., Brion, S., Moore, D. A. & Kennedy, J. A. A status-enhancement account of overconfidence. *J. Pers. Soc. Psychol.* **103**, 718-735 (2012).
24. Van Kleef, G. A., Homan, A. C., Finkenauer, C., Gündemir, S. & Stamkou, E. Breaking the rules to rise to power: how norm violators gain power in the eyes of others. *Soc. Psychol. Pers. Sci.* **2**, 500-507 (2011).
25. Koenig, A. M., Eagly, A. H., Mitchell, A. A. & Ristikari, T. Are leader stereotypes masculine? A meta-analysis of three research paradigms. *Psychol. Bull.* **137**, 616-642 (2011).
26. Ensari, N., Riggio, R. E., Christian, J. & Carslaw, G. Who emerges as a leader? Meta-analyses of individual differences as predictors of leadership emergence. *Pers. Indiv. Differ.* **51**, 532-536 (2011).
27. Epitropaki, O. & Martin, R. Implicit leadership theories in applied settings: factor structure, generalizability, and stability over time. *J. Appl. Psychol.* **89**, 293-310 (2004).

28. Gartzia, L. & Baniandrés, J. Are people-oriented leaders perceived as less effective in task performance? Surprising results from two experimental studies. *J. Bus. Res.* **69**, 508-516 (2016).
29. Zhang, Y., Waldman, D. A., Han, Y.-L. & Li, X.-B. Paradoxical leader behaviors in people management: antecedents and consequences. *Acad. Manage. J.* **58**, 538-566 (2015).
30. Fruhen, L. S., Watkins, C. D. & Jones, B. C. Perceptions of facial dominance, trustworthiness and attractiveness predict managerial pay awards in experimental tasks. *Leadership Quart.* **26**, 1005-1016 (2015).
31. Anderson, C. & Kilduff, G. J. Why do dominant personalities attain influence in face-to-face groups? The competence-signaling effects of trait dominance. *J. Pers. Soc. Psychol.* **96**, 491-503 (2009).
32. Fiske, S. T., Dupree, C. H., Nicolas, G. & Swencionis, J. K. Status, power, and intergroup relations: the personal is the societal. *Curr. Opin. Psychol.* **11**, 44-48 (2016).
33. Keller Hansbrough, T. in *Emerging Challenges in Business, Optimization, Technology, and Industry* (eds Tadj, L. & Garg, A. K.) 15-20 (Springer, Cambridge, 2018).
34. Heinberg, L. J., Thompson, J. K. & Stormer, S. Development and validation of the Sociocultural Attitudes Towards Appearance Questionnaire. *Int. J. Eat. Disorder* **17**, 81-89 (1995).
35. Thompson, J. K., van den Berg, P., Roehrig, M., Guarda, A. S. & Heinberg, L. J. The Sociocultural Attitudes Towards Appearance Scale-3 (SATAQ-3): development and validation. *Int. J. Eat. Disorder* **35**, 293-304 (2004).
36. Stice, E. Risk and maintenance factors for eating pathology: a meta-analytic review. *Psychol. Bull.* **128**, 825-848 (2002).
37. Teige-Mocigemba, S., Klauer, K. C. & Sherman, J. W. in *Handbook of Implicit Social Cognition: Measurement, Theory, and Applications* (eds Gawronski, B. & Payne, B. K.) 117-139 (Guilford Press, New York, 2010).
38. Greenwald, A. G., Poehlman, T. A., Uhlmann, E. L. & Banaji, M. R. Understanding and using the Implicit Association Test: III. meta-analysis of predictive validity. *J. Pers. Soc. Psychol.* **97**, 17-41 (2009).
39. Asch, S. E. Studies of independence and conformity: I. a minority of one against a unanimous majority. *Psychol. Monogr.-Gen. A.* **70**, 1-70 (1956).
40. Baron, R. S., Vandello, J. A. & Brunsman, B. The forgotten variable in conformity research: impact of task importance on social influence. *J. Pers. Soc. Psychol.* **71**, 915-927 (1996).
41. Nagel, R., Bühren, C. & Frank, B. Inspired and inspiring: Hervé Moulin and the discovery of the beauty contest game. *Math. Soc. Sci.* **90**, 191-207 (2017).
42. McClelland, D. C. & Boyatzis, R. E. Leadership motive pattern and long-term success in management. *J. Appl. Psychol.* **67**, 737-743 (1982).
43. Cornelius, E. T. & Lane, F. B. The power motive and managerial success in a professionally oriented service industry organization. *J. Appl. Psychol.* **69**, 32-39 (1984).
44. Sorrentino, R. M. & Field, N. Emergent leadership over time: the functional value of positive motivation. *J. Pers. Soc. Psychol.* **50**, 1091-1099 (1986).
45. Winter, D. G. Leader appeal, leader performance, and the motive profiles of leaders and followers: a study of American presidents and elections. *J. Pers. Soc. Psychol.* **52**, 196-202 (1987).
46. Spangler, W. D. & House, R. J. Presidential effectiveness and the leadership motive profile. *J. Pers. Soc. Psychol.* **60**, 439-455 (1991).
47. House, R. J., Spangler, W. D. & Woycke, J. Personality and charisma in the U.S. presidency: a psychological theory of leader effectiveness. *Admin. Sci. Quart.* **36**, 364-396 (1991).
48. Winter, D. G. A motivational model of leadership: predicting long-term management success from TAT measures of power motivation and responsibility. *Leadership Quart.* **2**, 67-80 (1991).
49. Winter, D. G. Power, affiliation, and war: three tests of a motivational model. *J. Pers. Soc. Psychol.* **65**, 532-545 (1993).
50. Jacobs, R. L. & McClelland, D. C. Moving up the corporate ladder: a longitudinal study of the leadership motive pattern and managerial success in women and men. *Consult. Psychol. J.: Pract. Res.* **46**, 32-41 (1994).
51. Langner, C. A. & Winter, D. G. The motivational basis of concessions and compromise: archival and laboratory studies. *J. Pers. Soc. Psychol.* **81**, 711-727 (2001).
52. Kirkpatrick, S. A., Wofford, J. C. & Baum, J. R. Measuring motive imagery contained in the vision statement. *Leadership Quart.* **13**, 139-150 (2002).
53. Schultheiss, O. C. & Brunstein, J. C. Inhibited power motivation and persuasive communication: a lens model analysis. *J. Pers.* **70**, 553-582 (2002).
54. De Hoogh, A. H. B. *et al.* Leader motives, charismatic leadership, and subordinates' work attitude in the profit and voluntary sector. *Leadership Quart.* **16**, 17-38 (2005).
55. Kazén, M. & Kuhl, J. Directional discrepancy between implicit and explicit power motives is related to well-being among managers. *Motiv. Emotion* **35**, 317-327 (2011).
56. Delbecq, A., House, R. J., de Luque, M. S. & Quigley, N. R. Implicit motives, leadership, and follower outcomes: an empirical test of CEOs. *J. Leadersh. Org. Stud.* **20**, 7-24 (2013).

57. Howard, A. in *New Developments in Goal Setting and Task Performance* (eds Locke, E. A. & G. Latham, P.) 246-261 (Routledge, New York, 2013).
58. Steinmann, B., Dörr, S. L., Schultheiss, O. C. & Maier, G. W. Implicit motives and leadership performance revisited: what constitutes the leadership motive pattern? *Motiv. Emotion* **39**, 167-174 (2015).
59. Steinmann, B., Ötting, S. K. & Maier, G. W. Need for affiliation as a motivational add-on for leadership behaviors and managerial success. *Front. Psychol.* **7**, 1972 (2016).
60. Winter, D. G. *The Power Motive* (The Free Press, New York, 1973).
61. Burnham, D. H. *Power is still the great motivator–with a difference!* Retrieved from https://pdfs.semanticscholar.org/9711/c9e46bcc07051806e9e5de78a1a0253bc553.pdf (1997).
62. Lukié, M. *Wie Motive den Managementerfolg beeinflussen*. Retrieved from https://www.manager-berater.com/assets/uploads/pdf/Wie-Motive-den-Managementerfolg-beeinflussen.pdf (2015).
63. Winter, D. G. What does Trump really want?. *Anal. Soc. Iss. Pub. Pol.* **18**, 155-171 (2018).
64. Jenkins, S. R. Need for power and women's careers over 14 years: structural power, job satisfaction, and motive change. *J. Pers. Soc. Psychol.* **66**, 155-165 (1994).
65. Winter, D. G., John, O. P., Stewart, A. J., Klohnen, E. C. & Duncan, L. E. Traits and motives: toward an integration of two traditions in personality research. *Psychol. Rev.* **105**, 230-250 (1998).
66. Winter, D. G. *Navy leadership and management competencies: convergence among tests, interviews and performance ratings* (McBer and Company, Boston, 1979).
